# Efficacy of Interval Training in Improving Body Composition and Adiposity in Apparently Healthy Adults: An Umbrella Review with Meta-Analysis

**DOI:** 10.1007/s40279-024-02070-9

**Published:** 2024-07-14

**Authors:** Eric Tsz-Chun Poon, Hong-Yat Li, Jonathan Peter Little, Stephen Heung-Sang Wong, Robin Sze-Tak Ho

**Affiliations:** 1 Department of Sports Science and Physical Education, The Chinese University of Hong Kong, Shatin, Hong Kong; 2 School of Health and Exercise Sciences, University of British Columbia, Kelowna, BC Canada; 3 Physical Education Unit, The Chinese University of Hong Kong, Shatin, Hong Kong

## Abstract

**Background:**

Although the efficacy of interval training for improving body composition has been summarized in an increasing number of systematic reviews in recent years, discrepancies in review findings and conclusions have been observed.

**Objective:**

This study aims to synthesize the available evidence on the efficacy of interval training compared with moderate-intensity continuous training (MICT) and nonexercise control (CON) in reducing body adiposity in apparently healthy adults.

**Methods:**

An umbrella review with meta-analysis was performed. A systematic search was conducted in seven databases (MEDLINE, EMBASE, Cochrane Database, CINAHL, Scopus, SPORTDiscus, and Web of Science) up to October 2023. Systematic reviews with meta-analyses of randomized controlled trials (RCTs) comparing interval training and MICT/CON were included. Literature selection, data extraction, and methodological quality assessment (AMSTAR-2) were conducted independently by two reviewers. Meta-analyses were performed using a random-effects model. Subgroup analyses were conducted based on the type of interval training [high-intensity interval training (HIIT) and sprint interval training (SIT)], intervention duration, body mass index, exercise modality, and volume of HIIT protocols.

**Results:**

Sixteen systematic reviews, including 79 RCTs and 2474 unique participants, met the inclusion criteria. Most systematic reviews had a critically low (*n* = 6) or low (*n* = 6) AMSTAR-2 score. Interval training demonstrated significantly greater reductions in total body fat percent (BF%) compared with MICT [weighted mean difference (WMD) of − 0.77%; 95% confidence interval (CI) − 1.12 to − 0.32%] and CON (WMD of − 1.50%; 95% CI − 2.40 to − 0.58%). Significant reductions in fat mass, visceral adipose tissue, subcutaneous abdominal fat, and android abdominal fat were also observed following interval training compared to CON. Subgroup analyses indicated that both HIIT and SIT resulted in superior BF% loss than MICT. These benefits appeared to be more prominent in individuals with overweight/obesity and longer duration interventions (≥ 12 weeks), as well as in protocols using cycling as a modality and low-volume HIIT (i.e., < 15 min of high-intensity exercise per session).

**Conclusions:**

This novel umbrella review with large-scale meta-analysis provides an updated synthesis of evidence with implications for physical activity guideline recommendations. The findings support interval training as a viable exercise strategy for reducing adiposity in the general population.

**Supplementary Information:**

The online version contains supplementary material available at 10.1007/s40279-024-02070-9.

## Key Points

**Table:** 

Interval training demonstrated a small but significantly greater reduction in total body fat percent (BF%) compared with moderate-intensity continuous training (MICT) and significant reductions in fat mass, visceral adipose tissue, subcutaneous abdominal fat, and android abdominal fat compared with nonexercise control.
Subgroup analyses indicated that both high-intensity interval training (HIIT) and sprint interval training (SIT) resulted in superior BF% loss versus MICT.
These benefits appeared to be more prominent in individuals with overweight/obesity and longer duration interventions (≥ 12 weeks), as well as in protocols using cycling as a modality and low-volume HIIT (i.e., < 15 min of high-intensity exercise per session).

## Introduction

The World Health Organization (WHO) defines excess weight and obesity as abnormal or excess fat accumulation that poses a risk to health. Obesity is an independent risk factor for various noncommunicable diseases, including heart disease, stroke, cancer, chronic respiratory diseases, and type 2 diabetes [1]. Regular physical activity (PA) and exercise play a crucial role in weight management by promoting calorie expenditure, enhancing metabolism, and supporting healthy body composition [1, 2].The current PA guidelines recommend that adults (18–65 years old) should engage in a minimum of 75–150 min of weekly moderate-to-vigorous physical activity (MVPA) to enhance health [2, 3], However, insufficient PA remains a prominent global issue [4]. Therefore, identifying effective, evidence-based, and practical exercise strategies aimed at mitigating the detrimental health consequences of physical inactivity and obesity has important clinical implications.

Recent bibliometric evidence has highlighted interval training as an emerging exercise strategy for improving health-related fitness in the general population compared with traditional training methods [5]. Interval training has attracted widespread attention among health and fitness professionals over the past decade and has been ranked among the top trends in the American College of Sports Medicine (ACSM) Worldwide Survey of Fitness Trends since 2013 [6]. Interval training typically involves repeated bouts of high-intensity exercise, interspersed with active or inactive periods of rest or recovery [7, 8]. Interval training is commonly classified as high-intensity interval training (HIIT) or sprint interval training (SIT) [2, 9], although various iterations appear in the literature. In a health context, HIIT can be characterized as intermittent bouts of exercise performed above moderate intensity (typically up to 4 min), primarily falling within the classification of vigorous intensity exercise (e.g., ~ 80–95% maximal heart rate) [8]. On the other hand, SIT represents a particularly intense variant of interval training that can be distinguished as repeated sprints at supramaximal intensities, typically performed with “all-out” efforts lasting ≤ 30 s [9].

Until recently, interval training has been recognized as an alternative option to traditional exercise approaches like moderate-intensity continuous training (MICT) in various authoritative PA guidelines worldwide, including those by the ACSM [2], the USA [10] and the United Kingdom [11]. However, it is important to note that these guidelines often lack a clear distinction between HIIT and SIT. For instance, the US guidelines mention the absence of universally accepted durations for the “maximal-effort” period, recovery period, ratio of the two, number of cycles per session, overall session duration, and the specific relative intensity at which the maximal-effort component should be performed during interval training [10]. Similarly, the UK guidelines state that data on HIIT are still emerging, and that further investigation is necessary to determine the optimal amount and form of HIIT to recommend [11]. These acknowledged limitations in authoritative guidelines underscore the need for additional research to comprehensively analyze the effectiveness of interval training, encompassing both HIIT and SIT, on body composition outcomes. Furthermore, while HIIT has received more attention in the literature and may be deemed more suitable for wider populations [7], SIT may still be considered a feasible option for relatively active and healthy individuals if appropriately designed [12, 13]. Therefore, including SIT in evaluating the overall efficacy of interval training allows for a broader range of interventions that are relevant and applicable to different populations.

While original studies exploring the efficacy of interval training in improving body adiposity in both general or populations with overweight/obesity have been conducted and summarized in an increasing number of systematic reviews in recent years, discrepancies in review findings and conclusions have been observed. For instance, some systematic reviews revealed the benefits of interval training in improving body composition, such as reducing whole-body fat and visceral fat when compared with MICT [14–16], whereas contrasting findings from other reviews have indicated a lack of significant differences [17, 18]. These systematic reviews, often focused on specific population subgroups (e.g., average healthy or individuals with overweight/obesity), interval training regimens (e.g., HIIT/SIT), intervention duration (e.g., short-term/long-term), comparator groups (e.g., MICT/ nonexercising control), or on specific anthropometric outcomes, pose challenges for healthcare professionals and researchers to understand the total body of evidence for interval training in the management of body fat reduction.

In this regard, umbrella reviews (also termed overviews of systematic reviews or meta-reviews) have been proposed as an effective approach to present a comprehensive overview of evidence synthesis on a given topic. Umbrella reviews summarize existing evidence from systematic reviews, making them a comprehensive means to inform guidelines. To the best of our knowledge, only one umbrella review has previously been conducted regarding the efficacy of interval training across the general population [19]. While that review suggested that interval training, in the form of HIIT, is effective and safe for improving cardiometabolic health and anthropometric measures, the results were described narratively without additional statistical analysis (i.e., quantitative meta-analysis). In addition, the article included systematic reviews of both randomized controlled trials (RCTs) and nonrandomized trials that mostly compared HIIT with an active control, but it did not report whether and how this form of interval training was superior to a nonactive control. High quality RCTs encompassing various forms of interval training and including both active and nonactive control groups would be required to provide further insights on the full range of benefits of interval training. Considering the substantial increase in evidence published from past systematic reviews and meta-analyses in recent years [20], an umbrella review that can address the aforementioned research gaps to further establish the benefits, compliance, and applications of interval training interventions among the general population appears timely. Therefore, we set out to undertake the most comprehensive synthesis of evidence to date regarding the effect of interval training on body composition and adiposity in adults.

## Methods

### Search Strategy

This overview of systematic reviews was performed in accordance with the Preferred Reporting Items for Overviews of Reviews (PRIOR) statement [21] and registered in the PROSPERO database (CRD42023490819). Seven databases were searched (MEDLINE, EMBASE, Cochrane Database, CINAHL, Scopus, SPORTDiscus, and Web of Science) using subject heading, keyword, and medical subject headings (MeSH) term searches for “systematic review,” “meta-analysis,” “HIIT,” and “body adiposity.” Database searches were limited to peer-reviewed systematic review articles published in English language from inception to 1 October 2023. The reference lists of the selected review articles were also examined for other potentially eligible papers. The detailed search strategy is presented in Supplementary Table 1.

### Selection Procedure and Eligibility Criteria

The population, interventions, comparators, outcomes, and study type (PICOS) framework was used to develop the inclusion criteria.

#### Types of Population

The population of interest was men and women aged 18 years or above, who were not suffering from any kind of acute or chronic disease, except for obesity. No exclusion criteria were applied to participants’ baseline fitness. Individuals who simultaneously have obesity and related comorbidities (e.g., cardiovascular diseases and type 2 diabetes) were excluded.

#### Types of Interventions

The term “interval training” has been used extensively in the literature to describe a variety of different high-intensity protocols that vary in the number and intensity of intervals, the time and nature (active or passive) of recovery periods, and total volume [8]; therefore, the definitions used in the present review are based on a general classification scheme for interval training put forward by Weston et al. [9]. The two most common protocols, HIIT and SIT, were differentiated based on the exercise intensity and unique characteristics observed in previous interval training studies. HIIT is generally defined as “near maximal” efforts performed at an intensity that elicits ≥ 80% maximal heart rate (HR_max_) or peak oxygen uptake, whereas SIT is characterized by repeated “all-out” sprints at supramaximal intensities (i.e., > 100% peak oxygen uptake) interspersed with recovery periods. Studies were eligible irrespective of interval training modality (e.g., treadmill running, cycling, or body-weight exercises), settings (e.g., clinical, laboratory, or community facility) or dose (frequency and duration).

#### Type of Comparators

In this overview of reviews, studies with no comparison groups were excluded. RCTs that involved MICT and/or nonexercise control (CON) comparison groups were included. MICT describes “traditional” exercise protocols performed continuously at a steady state for a set duration (usually 20–60 min) [9]. Moderate intensity is defined as intensity that induces a heart rate response of 60–79% HRmax or that elevates the rate of oxygen consumption to 40–59% of peak oxygen uptake [9].

#### Types of Outcomes

The results quantitatively reported from each embedded RCT included at least one of the following outcomes: total body fat percentage (BF%), body mass (BM), fat mass (FM), body mass index (BMI), waist circumference (WC), waist-to-hip ratio (WHR), lean mass (LM), fat-free mass (FFM), visceral adipose tissue (VAT), and abdominal fat (AF).

#### Types of Studies

Systematic reviews (with or without meta-analyses) of RCTs were selected.

### Selection of Literature and Data Extraction

Search results were imported into EndNote X10 (Clarivate, Philadelphia). Two reviewers (EP and JHL) independently screened the titles and abstracts of the retrieved citations from the seven electronic databases, removed duplicates, and determined eligible systematic reviews based on our inclusion criteria. For each eligible citation from our previous step, full texts of the embedded citations were obtained. Inter-reviewer disagreements were resolved by consensus or arbitration by a third reviewer (R.H.). Data from included systematic reviews were extracted in duplicate by two independent reviewers (E.P. and J.H.L.) using a standardized extraction form. The extracted data included the lead author, year of publication, design of original studies, population characteristics (age and sex), number of original studies, and participants included, description of interval training interventions (protocols, frequency, and duration), comparison groups, and outcomes.

Considering that some of the systematic reviews included might have contained certain component RCTs that did not meet our inclusion criteria (e.g., “contamination” of RCTs with ineligible participants, interventions, or outcomes), every component RCT from the included reviews was further screened by two reviewers (E.P. and J.H.L.) independently to ensure relevance. The inclusion criteria for the RCTs in the umbrella review remained consistent with the aforementioned criteria. Inter-reviewer disagreements were resolved by consensus or arbitration by a third reviewer (R.H.). Subsequently, data from eligible RCTs were extracted, including the first author, year of publication, characteristics of participants, and sample size. The intervention features were also extracted to assist the reviewers in subcategorizing the interval training.

### Critical Appraisal of Systematic Reviews and Randomized Controlled Trials

Critical appraisals of both systematic reviews and RCTs were independently performed by the two reviewers (E.P. and J.H.L.), and discrepancies were resolved through discussions. Discrepancies were resolved by consensus or arbitration by a third reviewer (R.H.).

#### Methodological Quality Assessment of Included Systematic Reviews

The methodological quality of the included reviews was assessed by two independent reviewers (E.P. and J.H.L.) in duplicate using the A MeaSurement Tool to Assess systematic Reviews (AMSTAR-2) tool [22]. The AMSTAR-2 tool involves 16 items, with each item scored as yes, partial yes or no. Seven items are considered “critical” and nine “noncritical” [22]. The critical domains are protocol registration, adequacy of search strategy, justification for excluding individual studies, risk of bias assessment, appropriateness of meta-analysis methods, use of risk of bias during interpretation, and assessment of publication bias. Reviews were rated as “high confidence” (zero critical weakness and less than three noncritical weaknesses), “moderate” (one critical weakness and less than three noncritical weaknesses), “low” (greater than one critical weakness and less than three noncritical weaknesses), or “critically low” (greater than one critical weakness and greater than or equal to three noncritical weaknesses) [22].

#### Methodological Quality Assessment of Included Randomized Controlled Trials

The methodological quality of the included RCTs was also independently assessed by two reviewers (E.P. and J.H.L.) using the modified physiotherapy Evidence Database (PEDro) scale. The original PEDro scale used an 11-point scale, but due to the impracticality of blinding participants and investigators in supervised exercise interventions, we opted to exclude assessment items related to blinding (numbers 5, 6, and 7 in the scale) as in previous exercise-related systematic reviews. Consequently, the modified 8-point PEDro scale has a maximum value of 7 (excluding the first item from the total score). The qualitative methodological ratings were adjusted as follows: “excellent” (6–7 points), “good” (5 points), “moderate” (4 points), and “poor” (0–3 points).

### Umbrella Review Synthesis Methods

The overlap in component RCTs that were included across all eligible reviews was assessed using the corrected covered area (CCA) method [23]. A CCA of 100% indicates that every review included in our umbrella review comprised the same component RCTs, while a CCA of 0% indicates that every review in our umbrella review included entirely unique RCTs. The following cutoffs were used to quantify the CCA: 0–5%, “slight overlap;” 6–10%, “moderate;” 11–15%, “high;” and > 15%, “very high” overlap [23].

Meta-analyses from eligible component RCTs were conducted using Review Manager software (RevMan 5.4; Cochrane Collaboration, Oxford, United Kingdom). The absolute change in mean difference and standard deviation of the outcome value from postintervention between groups (interval training versus MICT/CON) in each study was calculated and pooled using the random-effects model. For studies that compared multiple intervention groups with a single comparison group (or vice versa), the sample size of the shared comparison group was split to avoid double counting [24]. Weighted mean differences (WMDs) with 95% confidence intervals (CIs) were used to synthesize continuous outcomes and create forest plots, except for VAT and AF outcomes, where standardized mean differences (SMDs) were used. Subgroup analyses were conducted based on the type of interval training (HIIT or SIT), intervention duration (< 12 weeks or ≥ 12 weeks), body mass index (BMI 18.5–24.9 or ≥ 25 kg/m^2^), exercise mode (cycling or running/walking/jogging), and HIIT volume (< 15 min and ≥ 15 min of high-intensity exercise per session). The heterogeneity of included RCTs was assessed using the *I*^2^ statistic, in which values of < 25%, 50%, and 75% were considered indicative of low, moderate, and high heterogeneity, respectively. Inverse variance weighting was used to compensate for the heterogeneity of sample sizes between studies. Publication bias was assessed by creating a funnel plot and observing the presence of asymmetries or missing sections.

## Results

### Overview of Search Results

**Fig. 1:**
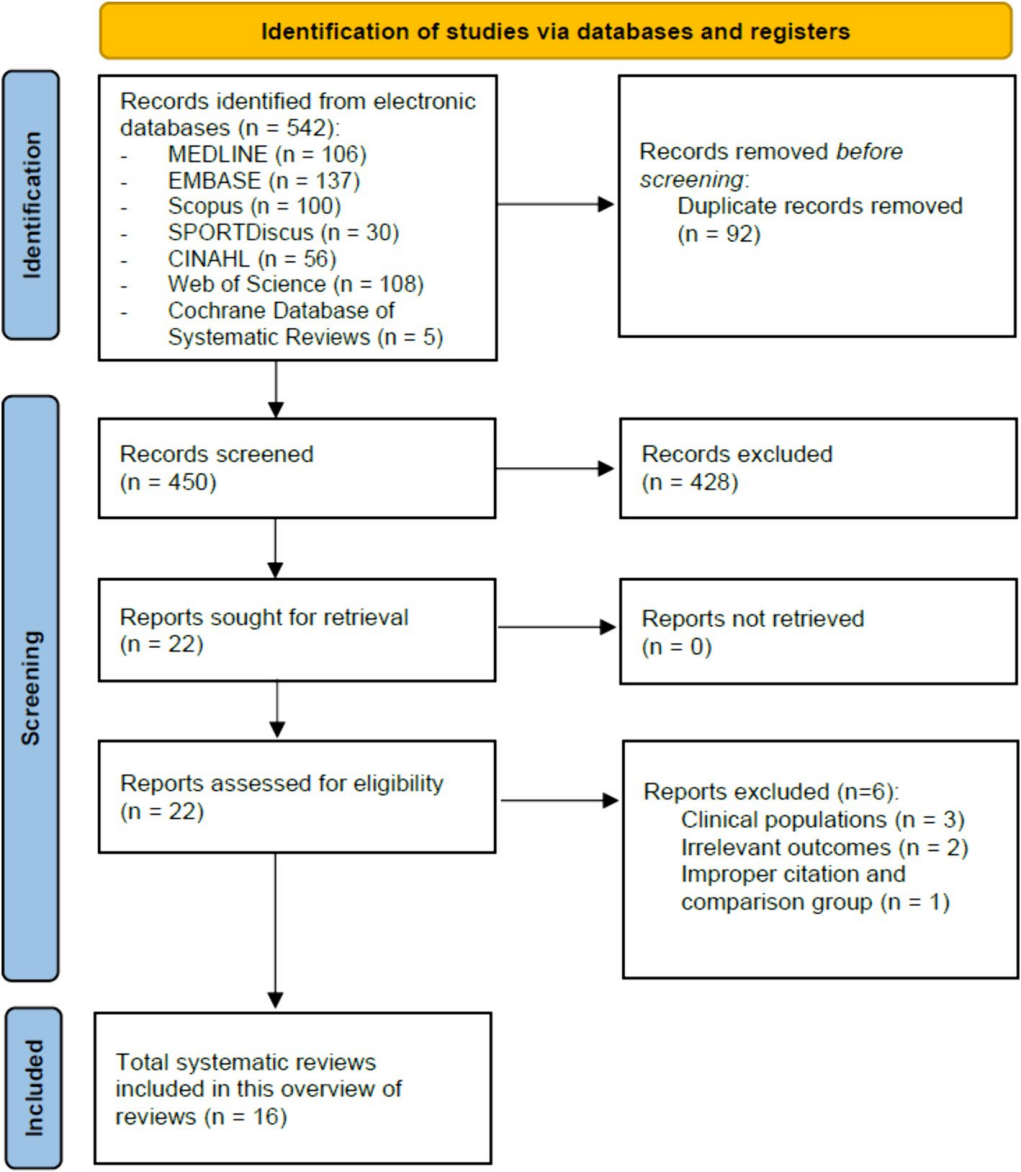
PRISMA flowchart of literature selection on systematic reviews

Out of the 542 records identified, 16 systematic reviews were included in this overview for the subsequent literature screening for eligible RCTs (see Fig. 1 for PRISMA flowchart). Table 1 provides an overview of the characteristics of all the reviews. A total of 432 original studies were listed in the included systematic reviews, with a CCA of 2.9%, indicating slight overlap.

**Table 1:** Summary of included systematic reviews

References	Population	Interventions and comparators	Measurement	Major outcomes
Alzar-Teruel et al. [104]	Original studies included: 8 Sample size: 615 Drop out: 152 (24.7%) Age: > 55 years Male (*n* = 215), female (*n* = 400)	*Intervention no. 1: HIIT* Duration: 6–24 weeks Frequency: 1–5 times per week Intensity^a^: > 70% VO_2max_, all out, RPE 14–18, 90–95% HRmax Mode: resistance training, circuit exercise, walking, conditioning exercise *Control no. 1: other exercise* MIIT, MICT, traditional resistance exercise, low-moderate intensity walking *Control no. 2: nonexercise*	Whole-body DEXA, BIA, electronic scale, and height rod	HIIT has beneficial effects on body composition, but it is unclear to determine whether HIIT is more effective than other type of training (i.e., MIIT, MICT, or traditional resistance training) among middle-aged and older adults
Andreato et al. [130]	Original studies included: 48 Sample size: 1222 Drop out^a^: 155 (12.7%) Age: 18–65 years Sex of original studies: mixed (37.3%), Men only (22%), women only (39%), not differentiated (1.7%)	*Intervention no. 1: HIIT* (*n* = 678) Duration: 2–24 weeks Frequency: 3–5 times per week Intensity: $\ge$ 80% VO_2max_ / > 80% HRR > 85% HR_max_ Mode: cycling, running, walking *Control no. 1: MICT* (*n* = 293) *Control no. 2: nonexercise* (*n* = 251)	DEXA, BIA, skinfold, CT, MRI, plethysmography	HIIT and MICT are similar, so HIIT can be secondary method for treating obese adults In normal weight, only VO2_max_ was significantly improved in both HIIT, while no other effect was observed. However, HIIT was effective for overweight/obese populations
Batacan et al. [131]	Original studies included: 65 Sample size: 2165 Drop out: NR Age: 18–35 years Sex: NR Normal weight/overweight/obsess	*Intervention no. 1: short-term (< 12 weeks) HIIT* (*n* = 473) Duration: 30 min to 10 weeks Frequency: 1–5 times per week Intensity: $\ge$ 85% VO_2max_/$\ge$ 85% HRR/$\ge$ 90% HR_max_ Mode: treadmill running, cycling *Intervention no. 2: long-term (*$\ge$* 12 weeks) HIIT* (*n* = 462) Duration: 12–52 weeks Frequency: 2–5 times per week Intensity: $\ge$ 85% VO_2max_/$\ge$ 85% HRR/$\ge$ 90% HR_max_ Mode: treadmill running, cycling, swimming *Other: NR* (*n* = 1230)	NR	
Chang et al. [16]	Original studies included: 34 Sample size: 1962 Drop out: NR Age: 18–75 years Male (*n* = 645), female (*n* = 1243), not differentiated (*n* = 74) BMI: 22–40.3 kg/m^2^	*Intervention no. 1: HIIT* (*n* = 174) Duration: 4–16 weeks Frequency: 2–5 times per week Intensity^a^: RPE 16–17, 77–95% HR_max_, 70–100% VO_2max_ Mode: treadmill, walking, TRX and body weight exercise, running, cycling *Intervention no. 2: SIT* (*n* = 46) Duration: 12 weeks Frequency: 3 times per week Intensity: all-out Mode: cycling *Control no. 1: AE* (*n* = 801) *Control no. 2: RE* (*n* = 204) *Control no. 3: AE and RE* (*n* = 128) *Control no. 4: nonexercise* (*n* = 609)	CT, MRI, DEXA, ultrasound, BIA	Adults can choose HIIT or AE with at least moderate intensity for reducing VF
Depiazzi et al. [132]	Original studies included: 8 Sample size: 377 Drop out: NR Age: 21.7–69.8 years Male (*n* = 41), female (*n* = 336)	*Intervention no. 1: HIIT* (*n* = 212) Duration: 8–24 weeks Frequency: 2–3 times per week Intensity: > 75% HR_max_, RPE > 15, > 75% VO_2max_, all-out Mode: aquatic *Control no. 1: nonexercise* (*n* = 165)	Skinfold, DEXA, ADP	No differences were seen in measures of body composition
Guo et al. [133]	Original studies included: 9 Sample size: 230 Drop out: NR Age: $\ge$ 18 years Sex: NR	*Intervention no. 1: HIIT + fasting* (*n* = 124) Duration: 13 days to 1 year Frequency: 3–7 times per week Intensity: 90% HR_max_, 85–90% VO_2max_, all-out Mode: cycling *Control no. 1: HIIT alone/fasting alone/normal diet and exercise* (*n* = 106)	Hydrostatic weighting, DEXA, BIA, CT, MRI	HIIT combined with fasting can effectively reduce body mass, BMI, WC, and BF of adults with overweight and obesity
Hwang et al. [134]	Original studies included: 6 Sample size: 162 Drop out: NR Age: 40.9–76.5 years Male (*n* = 100), female (*n* = 62)	*Intervention no. 1: HIIT* (*n* = 77) Duration: 4–16 weeks Frequency: 3–5 times per week Intensity: 80–105% VO_2peak_, 85–95% HR_max_ Mode: running, cycling *Control no. 1: MICT* (*n* = 76) *Control no. 2: usual care* (*n* = 9)	NR	HIIT has similar benefits on metabolic risk as MICT. It may be used in suitable patients with cardiometabolic disorders but just based on a few randomized controlled trials
Keating et al. [135]	Original studies included: 31 Sample size: 840 Drop out: NR Age: 10.1–65 years Male (*n* = 410), female (*n* = 402), not differentiated (*n* = 28)	*Intervention no. 1: HIIT* (*n* = 257) Duration: 4–16 weeks Frequency: 2–5 times per week Intensity: 80–100% HR peak Mode: running, jogging, walking, cycling, skipping and boxing drills, all-extremity air-baked ergometer *Intervention no. 2: SIT* (*n* = 167) Duration: 4–15 weeks Frequency: 3–4 times per week Intensity: > 100% VO_2max_ Mode: cycling, running, swimming *Control no. 1: MICT* (*n* = 416)	Hydrodensitometry, DEXA, whole body densitometry via air displacement, BIA, 2/4/6/7/8 sites skinfold, MRI, CT	HIIT is similar to MICT; both significantly reduced total body fat and fat mass
Rugbeer et al. [18]	Original studies included: 26 Sample size: 784 Drop out: NR Age: 17–69 years Sex: female > male (exact number: NR)	*Intervention no. 1: HIIT* (*n* = 292) Duration: 2–15 weeks Frequency: 3–5 times per week Intensity: $\ge$ 60% VO_2_R/HRR/$\ge 77$% HR_max_ Mode^a^: cycling, walking, jogging *Intervention no. 2: SIT* (*n* = 130) Duration: 2–12 weeks Frequency: 3 times per week Intensity: all-out Mode: cycling *Control no. 1: MICT* (*n* = 362)	NR	There was no significant difference in body fat percentage between MICT versus HIIT and SIT in people with overweight or obesity
Serrablo-Torrejon et al. [136]	Original studies included: 10 Sample size: 529 Drop out: NR Age: NR Sex of original studies: mixed (*N* = 8, 80%), men only (*N* = 1, 10%), women only (*N* = 1, 10%)	*Intervention no. 1: HIIT* (*n* = 355) Duration: 3–24 weeks Frequency: 3–5 times per week Intensity: Mode: cycling, running, walking *Control no. 1: nonexercise* (*n* = 174)	NR	HIIT showed significant physiological benefits for WC reduction
Steele et al. [137]	Original studies included: 54 Sample size: 1829 Drop out: HIIT (16.1%), MICT (20.1%) Age: 7–79 years Sex: NR	*Intervention no. 1: interval training* (not differentiated HIIT and SIT) (*n* = 830) Duration: 4–52 weeks Frequency: 2–5 times per week Intensity: > 75% HR_max_ Mode^a^: cycling, boxing, running, rowing *Control no. 1: MICT* (*n* = 773) *Control no. 2: nonexercise* (*n* = 226)	DEXA, BIA, skinfolds, hydrostatic, ADP	The patterns of intensity of effort and duration during endurance exercise have minimal influence on longitudinal changes in fat mass and fat free mass
Sultana et al. [17]	Original studies included: 47 Sample size: 1458 Drop out: NR Age: 19–70 years (mean) Male* (*n* = 661), female* (*n* = 753)	*Intervention no. 1: low-volume HIIT* ($\le$ 500 MET-min per week) (included HIIT and SIT protocol) (*n* = 662)^a^ Duration: 4–16 weeks Frequency: 2–5 times per week Intensity: 75–100% HR_max_, 80–170% VO_2max_, 60–140% Peak power output, all-out Mode: cycling, running, walking *Control no. 1: MICT* (*n* = 399)^a^ *Control no. 2: nonexercise* (*n* = 340)^a^	DEXA, BIA, ADP	No clear evidence that low-volume HIIT is superior to non-exercise control or MICT for improving total body fat mass, body fat percentage, lean body mass
Wang et al. [138]	Original studies included: 15 Sample size: 1134 Drop out: NR Age: 7–23 years Sex: NR	*Intervention no. 1: HIIT and SIT* (*n* = 109) Duration: 12 weeks Frequency: 3–4 times per week Intensity: all-out, 90–120% VO_2max_ Mode: cycling, running, walking *Intervention no. 2: AE* (*n* = 472) *Intervention no. 3: RE* (*n* = 87) *Intervention no. 4: AE + RE* (*n* = 83) *Control no. 1: NR* (*n* = 383)	NR	AE and HIIT have a significant effect on decreasing visceral fat and HIIT appears to be the most effective
Wang et al. [139]	Original studies included: 38 Sample size: 1317 (reported), 1330 (count) Drop out: NR Age: 10.8–69.5 years (mean) Male (*n* = 509), female (*n* = 740), not differentiated (*n* = 81)	*Intervention no. 1: HIIT* (*n* = 111) Duration: 6–16 weeks Frequency: 3–4 times per week Intensity: > 80% HR_max_, 75–80% HRR, 90–100% VO_2max_, maximal Mode^a^: running, cycling *Intervention no. 2: AE* (*n* = 448) *Intervention no. 3: RE* (*n* = 171) *Intervention no. 4: AE + RE* (*n* = 147) *Control no. 1: NR* (*n* = 453)	NR	Exercise intervention could effectively improve body composition
Wewege et al. [14]	Original studies included: 13 Sample size: 424 Drop out^a^: 29 Age: 31.9 years (mean) BMI: 29.7 kg/m^2^ (mean) Male (*n* = 212), female (*n* = 212)	*Intervention no. 1: HIIT* (*n* = 216) Duration: 5–16 weeks Frequency: 3–5 times/week Intensity: > 85% HR_max_, > 80% VO_2max_, RPE > 17 Mode: cycling, running *Control no. 1: MICT* (*n* = 208)	BIA, DEXA, CT	HIIT shows similar efficacy to MICT but with 40% less time commitment each week
Wu et al. [15]	Original studies included: 29 Sample size: 11,156 Drop out: NR Age: 61.1–84 years (mean) Male (*n* = 357), female (*n* = 468), not differentiated (*n* = 237)	*Intervention#1: HIIT* (*n* = 553) Duration: 4–24 weeks Frequency: 2–5 times per week Intensity: 80–95% HR_max_, $\ge$ 70% VO_2max_, > 90% HRR, 60–124% peak power output, RPE 11–13, 70% of 1 RM Mode: elliptical devices, cycling, circuit-based interval exercise, Xbox 360 s *Control no. 1: MICT* (*n* = 555)	DEXA, anthropometry measurement	HIIT was found to be a feasible and effective method to improve body composition

*ADP* air displacement plethysmography, *AE* aerobic exercise, *AF* abdominal fat, *ASF* abdominal subcutaneous fat, *BF*% body fat percentage, *BIA* bioelectrical impedance analysis, *BM* body mass, *BMI* body mass index, *CT* computer tomography, *DEXA* dual-energy X-ray absorptiometry, *FM* fat mass, *FatOX* fat oxidation, *HIIT* high-intensity interval training, *HR*_*max*_ maximal heart rate, *HRR* heart rate reserve, *LBM* lean body mass, *MICT* moderate-intensity continuous training, *MIIT* moderate-intensity interval training, *MRI* magnetic resonance imaging, *NR* not reported, *PRT* progressive resistance training, *PRT* progressive resistance training, *RE* resistance exercise, *RPE* rate of perceived exertion, *SIT* sprint interval training, *VF* visceral fat, *VO*_*2max*_ maximal oxygen uptake, *WC* waist circumference, *WHR* waist-to-hip ratio

^a^Included the reported data only

**Fig. 2:**
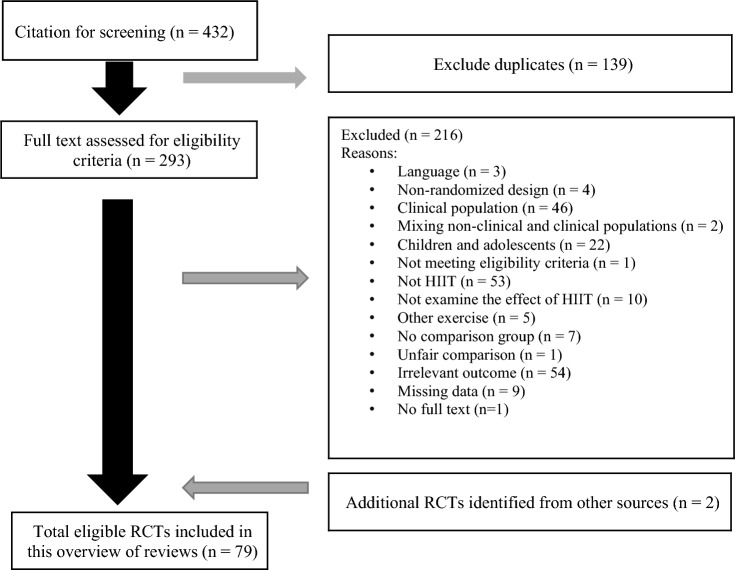
Flowchart for eligible randomized controlled trials selection for effects of interval training on body composition and adiposity outcomes

Of the 432 embedded RCTs, we excluded 139 duplicates and 216 studies (see Fig. 2 for flowchart and reasons for exclusions in Supplementary Table 4). A total of 77 eligible RCTs met our inclusion criteria from the included systematic reviews. Two additional RCTs were identified by checking the reference lists, resulting in 79 RCTs included in our overview for data extraction. Supplementary Table 2 summarizes the characteristics of all included studies [25–103]. Evaluation of funnel plots showed no evidence of publication bias. The majority of the original studies specifically involved individuals with overweight/obesity based on a BMI ≥ 25 kg/m^2^ (*N* = 57), while others involved participants with a BMI of 18–24.9 kg/m^2^ (*N* = 11), and a small number did not report (*N* = 6) or classify (*N* = 5) BMI.

### Participant Characteristics

The meta-analysis included a total of 2474 unique participants. They were assigned to the following groups: HIIT (*n* = 717), SIT (*n* = 485), MICT (*n* = 755), and CON (*n* = 517). Among the original studies that reported participants’ sex, the male (*n* = 1179) and female (*n* = 1225) ratio was similar. Among the RCTs that reported drop-out, a total of 376 participants (~ 15%) who initially enrolled in the studies were not included in the data analysis. In accordance with the inclusion criteria, participants were apparently healthy adults without acute or chronic diseases, with a mean age range of 18 to 73.5 years.

### Intervention Characteristics

The intervention characteristics of eligible RCTs are summarized in Supplementary Table 2. The duration of interventions ranged from 13 days to 16 weeks, with 29 studies lasting ≥ 12 weeks and 50 studies lasting < 12 weeks. The interventions generally had a frequency of 2–7 days per week, with each session lasting 8–70 min. Various exercise modalities were used in interval training, including cycling, running, aquatic treadmill running, all-extremity air-baked ergometer, circuit strength training, TRX, boxing drills (heavy bag, focus mitts, circular body weight footwork drills, skipping), circuit-based dynamic body-weight exercise, and walking/ jogging. The total set/bouts in the interval training protocols ranged from 2 to 80 times. Both passive and active recovery protocols were employed, with work-to-rest ratio ranging from 2:1 to 1:9.

### Methodological Quality of Included Reviews

Table 2 provides a summary of the AMSTAR-2 scores. The majority of the reviews had a critically low (*n* = 6) or low (*n* = 6) score, while two reviews had a moderate score. Specifically, only 37.5% of reviews referred to a predefined methodology (item 2). None of the studies provided a list of excluded studies with reasons for exclusions (item 7) or reported on the sources of funding for the included studies (item 10). All studies accounted for risk of bias (RoB) when interpreting the results (item 9) and 69% discussed heterogeneity (item 14). Among the reviews that conducted a meta-analysis, all used appropriate/partly appropriate methods for statistical combination of results (item 11), and 60% investigated publication bias (item 15). However, only one review (8%) assessed the impact of RoB on the results (item 12).

**Table 2:** AMSTAR-2 ratings of methodological quality of systematic reviews and meta-analyses

References	1	2	3	4	5	6	7	8	9	10	11	12	13	14	15	16	Confidence
Alzar-Teruel et al. [104]	N	N	Y	PY	Y	N	N	PY	Y	N	NA	NA	N	N	NA	Y	Critically low
Andreato et al. [130]	Y	Y	Y	PY	Y	Y	N	Y	Y	N	Y	N	Y	N	N	Y	Low
Batacan et al. [131]	Y	N	Y	PY	Y	N	N	PY	Y	N	Y	N	N	N	N	N	Critically low
Chang et al. [16]	Y	N	Y	PY	Y	N	N	PY	Y	N	Y	N	Y	Y	Y	Y	Low
Depiazzi et al. [132]	Y	Y	Y	PY	Y	Y	N	PY	Y	N	Y	N	Y	Y	N	Y	Low
Guo et al. [133]	N	Y	Y	PY	Y	Y	N	PY	Y	N	Y	N	Y	Y	Y	Y	Moderate
Hwang et al. [134]	Y	N	Y	PY	Y	N	N	PY	Y	N	Y	N	N	Y	N	Y	Critically low
Keating et al. [135]	Y	N	Y	PY	N	Y	N	PY	Y	N	Y	Y	Y	Y	Y	N	Low
Rugbeer et al. [18]	Y	N	Y	N	Y	N	N	PY	Y	N	Y	N	N	Y	N	N	Critically low
Serrablo-Torrejon et al. [136]	Y	N	Y	PY	Y	N	N	PY	Y	N	Y	N	N	N	N	Y	Critically low
Steele et al. [137]	Y	Y	Y	PY	Y	Y	N	PY	Y	N	Y	N	Y	Y	Y	Y	Moderate
Sultana et al. [17]	Y	N	Y	PY	Y	Y	N	PY	Y	N	Y	N	Y	Y	Y	Y	Low
Wang et al. [138]	Y	Y	Y	PY	Y	Y	N	PY	Y	N	Y	N	Y	Y	Y	Y	Moderate
Wang et al. [139]	Y	Y	Y	PY	Y	Y	N	PY	Y	N	Y	N	Y	Y	Y	Y	Moderate
Wewege et al. [14]	Y	N	Y	PY	Y	Y	N	PY	Y	N	Y	N	Y	Y	Y	Y	Low
Wu et al. [15]	N	N	Y	PY	Y	N	N	PY	Y	N	Y	N	N	N	Y	Y	Critically low

*N* no, *NA* not applicable (no meta-analysis), *PY* partial yes, *Y* yes

### Methodological Quality of Included RCTs

Supplementary Table 3 provides a summary of the PEDro scores. Among the 79 RCTs, the mean rating was 5.1, indicating that the overall collection of studies was of good quality. Of these, 33 studies were rated as excellent, 26 studies were rated as good, and 20 studies were rated as fair. Notably, none of the studies included in the analysis were deemed to be of poor quality.

### Meta-Analysis

#### Interval Training Versus CON

**Fig. 3:**
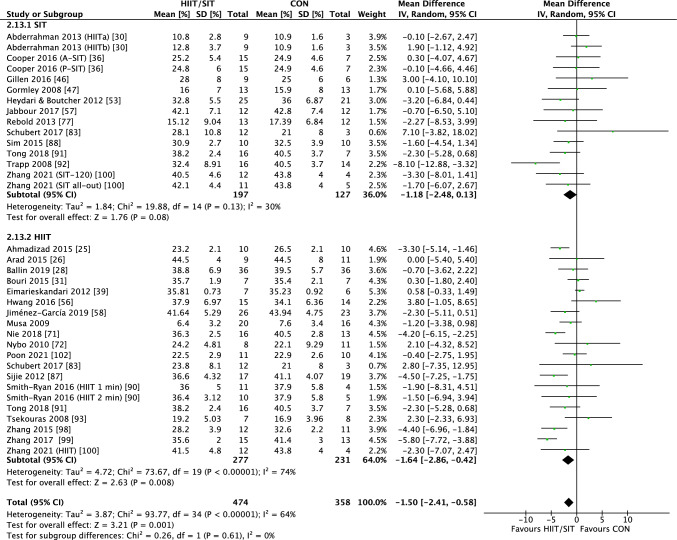
Forest plot for the between-group effects of interval training (HIIT/SIT) versus CON on body fat percent reduction. *CON* nonexercise control, *HIIT* high-intensity interval training, *SIT* Sprint interval training

The summary of meta-analyses is presented in Table 3. Compared with CON, interval training demonstrated significant reductions in total BF% (28 RCTs, WMD of − 1.50%; 95% CI − 2.41% to − 0.58%, *p* = 0.001, Fig. 3), FM (19 RCTs, WMD of − 0.79 kg; 95% CI − 1.55 to − 0.04 kg, *p* = 0.03), VAT (7 RCTs, SMD of − 0.26; 95% CI − 0.51 to − 0.01, *p* = 0.04), AF_subcutaneous_ (6 RCTs, SMD of − 0.33; 95% CI − 0.64 to − 0.02, *p* = 0.04), AF_android_ (4 RCTs, SMD of − 0.49; 95% CI − 0.90 to − 0.08, *p* = 0.02), and AF_gynoid_ (4 RCTs, SMD of − 1.26; 95% CI − 2.31 to − 0.21, *p* = 0.02). No significant between-group difference was observed for other outcome measures. Subgroup analyses indicated that longer duration interventions tended to result in greater reductions in BF%, BM, BMI, and FM. In addition, greater BF% loss was observed in individuals with overweight/obesity. Both HIIT (19 RCTs, WMD of − 1.64%; 95% CI − 2.86% to − 0.42%, *p* = 0.01) and SIT (12 RCTs, WMD − 1.81%; 95% CI − 2.48% to 0.13%, *p* = 0.08) showed similar reductions in BF% loss, whereas HIIT tended to favor reductions in BMI (18 RCTs, WMD of − 0.79 kg/m^2^; 95% CI − 1.52 to − 0.07 kg/m^2^, *p* = 0.03) compared with SIT (8 RCTs, WMD of 0.17 kg/m^2^; 95% CI − 1.13 to 0.80 kg/m^2^, *p* = 0.74). Cycling exercise mode (15 RCTs, WMD of − 1.63%; 95% CI − 2.97% to − 0.29%, *p* = 0.02) and low-volume HIIT (8 RCTs, WMD of − 1.62%; 95% CI − 2.71% to − 0.54%, *p* = 0.003) appeared to have more pronounced BF% reduction than running/walking/jogging (10 RCTs, WMD of − 0.90%; 95% CI − 2.25% to 0.45%, *p* = 0.19) and high-volume HIIT (6 RCTs, WMD of − 0.68%; 95% CI − 2.96% to 1.61%, *p* = 0.56).

**Table 3:** Summary of meta-analyses of interval training versus nonexercise control

Outcome	*N*	Mean difference (95% of CI)	*p*	*I*^2^ (*p*)
*Body fat (%)*	28	− 1.50 (− 2.41 to − 0.58)	**0.001**	64% (< 0.00001)
Protocol: SIT	12	− 1.81 (− 2.48 to 0.13)	0.08	30% (0.13)
Protocol: HIIT	19	− 1.64 (− 2.86 to − 0.42)	**0.008**	74% (< 0.00001)
Duration: long term	16	− 2.71 (− 3.73 to − 1.65)	** < 0.00001**	43% (0.02)
Duration: short term	12	− 0.12 (− 1.15 to 0.90)	0.81	39% (0.06)
BMI: 18.5–24.9 kg/m^2^	4	− 1.14 (− 3.76 to 1.47)	0.39	68% (0.01)
BMI: $\ge$ 25 kg/m^2^	20	− 1.66 (− 2.78 to − 0.54)	**0.004**	68% (< 0.00001)
Mode: cycling	15	− 1.63 (− 2.97 to − 0.29)	**0.02**	46% (0.01)
Mode: run/walk/jog	10	− 0.90 (− 2.25 to 0.45)	0.19	73% (< 0.0001)
HIIT volume: low	8	− 1.62 (− 2.71 to − 0.54)	**0.003**	0% (0.45)
HIIT volume: high	6	− 0.68 (− 2.96 to 1.61)	0.56	81% (< 0.0001)
*Body mass (kg)*	31	− 0.67 (− 1.92 to 0.58)	0.29	34% (0.02)
Protocol: SIT	11	− 0.20 (− 2.42 to 2.02)	0.86	34% (0.86)
Protocol: HIIT	22	− 0.89 (− 2.44 to 0.67)	0.26	38% (0.04)
Duration: long term	18	− 1.88 (− 3.50 to − 0.25)	**0.02**	21% (0.18)
Duration: short term	13	0.65 (− 1.19 to 2.489)	0.49	41% (0.05)
BMI: 18.5–24.9 kg/m^2^	5	1.25 (− 2.66 to 5.15)	0.53	59% (0.03)
BMI: $\ge$ 25 kg/m^2^	24	− 1.10 (− 2.53 to 0.33)	0.13	34% (0.04)
Mode: cycling	19	− 1.67 (− 3.02 to − 0.32)	**0.02**	0% (0.68)
Mode: run/walk/jog	12	0.41 (− 1.82 to 2.64)	0.52	64% (0.0006)
HIIT volume: low	9	1.61 (− 2.12 to 5.35)	0.40	59% (0.01)
HIIT volume: high	7	− 1.26 (− 3.45 to 0.94)	0.26	35% (0.16)
*Body mass index (kg/m*^*2*^*)*	25	− 0.53 (− 1.07 to 0.02)	0.06	71% (< 0.00001)
Protocol: SIT	8	− 0.17 (− 1.13 to 0.80)	0.74	72% (< 0.00001)
Protocol: HIIT	18	− 0.79 (− 1.52 to − 0.07)	**0.03**	70% (< 0.00001)
Duration: long term	12	− 1.20 (− 2.27 to − 0.13)	**0.03**	84% (< 0.00001)
Duration: short term	14	− 0.29 (− 0.53 to − 0.06)	**0.01**	0% (0.49)
BMI: 18.5–24.9 kg/m^2^	4	0.11 (− 0.95 to 1.17)	0.84	45% (0.12)
BMI: $\ge$ 25 kg/m^2^	21	− 0.77 (− 1.48 to − 0.05)	**0.04**	73% (< 0.0001)
Mode: cycling	13	− 0.34 (1.17 to 0.49)	0.42	64% (0.0004)
Mode: run/walk/jog	10	− 0.50 (− 1.23 to 0.24)	0.19	61% (0.005)
Protocol: SIT	4	− 0.61 (− 1.77 to 0.54)	0.30	0% (0.72)
Protocol: HIIT	6	− 1.36 (− 6.44 to 3.72)	0.18	66% (0.007)
HIIT volume: low	7	− 0.62 (− 1.52 to 0.29)	0.07	82% (< 0.00001)
HIIT volume: high	11	− 2.27 (− 4.73 to 0.18)	0.06	81% (< 0.0001)
*Waist circumference (cm)*	6	− 2.81 (− 5.68 to 0.07)	0.60	86% (< 0.00001)
*Waist-to-hip ratio*	7	− 0.02 (− 0.06 to 0.02)	0.23	95% (< 0.00001)
*Lean mass (kg)*	8	0.60 (− 0.49 to 1.70)	0.28	0% (0.66)
*Fat-free mass (kg)*	8	− 0.07 (− 1.39 to 1.26)	0.92	0% (0.77)
Protocol: SIT	4	1.43 (− 1.28 to 4.13)	0.30	0% (0.85)
Protocol: HIIT	5	− 0.54 (− 2.07 to 0.99)	0.49	0% (0.77)
*Fat mass (kg)*	19	− 0.79 (− 1.55 to − 0.04)	**0.04**	14% (0.26)
Protocol: SIT	6	− 1.81 (− 3.97 to 0.34)	0.10	0% (0.71)
Protocol: HIIT	17	− 0.73 (− 1.61 to 0.14)	0.10	26% (0.14)
Duration: long term	10	− 2.82 (− 4.13 to − 1.52)	** < 0.0001**	0% (0.71)
Duration: short term	9	− 0.01 (− 0.67 to 0.65)	0.98	0% (0.90)
Mode: cycling	12	− 2.20 (− 3.46 to − 0.95)	**0.0006**	0% (0.71)
Mode: run/walk/jog	5	− 0.14 (− 1.11 to 0.84)	0.78	34% (0.18)
HIIT volume: low	6	− 0.10 (− 0.88 to 0.69)	0.81	0% (0. 75)
HIIT volume: high	5	0.16 (− 1.84 to 2.16)	0.87	41% (0.15)
*Visceral adipose tissue*^a^	7	− 0.26 (− 0.51 to − 0.01)	**0.04**	0% (0.90)
*Abdominal fat (total)*^a^	5	− 0.29 (− 0.60 to 0.01)	0.06	0% (0.89)
*Abdominal fat (subcutaneous)*^a^	6	− 0.33 (− 0.64 to − 0.02)	**0.04**	8% (0.37)
*Abdominal fat (android)*^a^	4	− 0.49 (− 0.90 to − 0.08)	**0.02**	17% (0.30)
*Abdominal fat (gynoid)*^a^	4	− 1.26 (− 2.31 to − 0.21)	**0.02**	83% (< 0.00001)

*BMI* body mass index, *CON* nonexercise control, *HIIT* high-intensity interval training, *SIT* sprint interval training

^a^Reported as standardized mean difference; bold text signifies statistically significant results

#### Interval Training Versus MICT

**Fig. 4:**
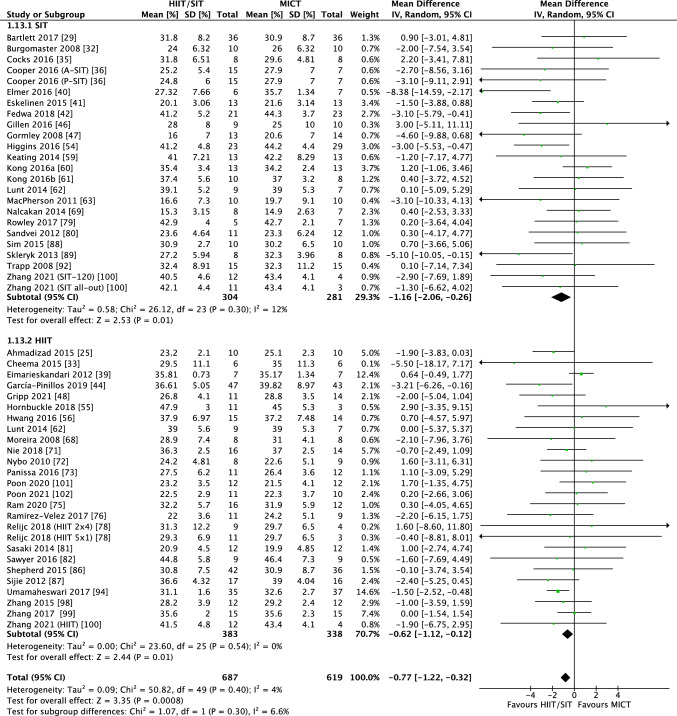
Forest plot for the between-group effects of interval training (HIIT/SIT) versus MICT on body fat percent reduction. *HIIT* high-intensity interval training, *MICT* moderate-intensity continuous training, *SIT* sprint interval training

The summary of meta-analyses is presented in Table 4. Compared with MICT, interval training demonstrated significantly greater reductions in total BF% (40 RCTs, WMD of − 0.77%; 95% CI − 1.22% to − 0.32%, *p* = 0.0008, Fig. 4). No significant between-group difference was observed for other outcome measures. Subgroup analyses indicated that both HIIT (25 RCTs, WMD of − 0.62%; 95% CI − 1.12% to − 0.12%, *p* = 0.01) and SIT (22 RCTs, WMD of − 1.16%; 95% CI − 2.06% to − 0.26%, *p* = 0.01) resulted in superior BF% loss than MICT (Fig. 4). Long-term interval training interventions (22 RCTs, WMD of − 1.10%; 95% CI − 1.67% to − 0.53%, *p* = 0.0002) and individuals with overweight/obesity (37 RCTs, WMD of − 0.74%; 95% CI − 1.19 to − 0.30%, *p* = 0.001) tended to show superior benefits of BF% loss than short-term interval training interventions (24 RCTs, WMD of − 0.38%; 95% CI − 1.22% to 0.46%, *p* = 0.38) and individuals with normal BMI (4 RCTs, WMD of − 0.45%; 95% CI − 2.87% to 1.97%, *p* = 0.72). Cycling exercise mode (29 RCTs, WMD of − 0.90%; 95% CI − 1.43% to − 0.36%, *p* = 0.001) and low-volume HIIT (11 RCTs, WMD − 1.14%; 95% CI − 1.94% to − 0.35%, *p* = 0.005) also appeared to have more pronounced BF% reduction than running/walking/jogging (14 RCTs, WMD of − 0.66%; 95% CI − 1.71% to 0.38%, *p* = 0.21) and high-volume HIIT (7 RCTs, WMD of − 0.03%; 95% CI − 0.99% to 0.92%, *p* = 0.94), when compared with MICT.

**Table 4:** Summary of meta-analyses of interval training versus moderate-intensity continuous training

Outcome	*N*	Mean difference(95% of CI)	*p*	*I*^2^ (*p*)
*Body fat (%)*	40	− 0.77 (− 1.22 to − 0.32)	**0.0008**	4% (0.40)
Protocol: SIT	22	− 1.16 (− 2.06 to − 0.26)	**0.01**	12% (0.30)
Protocol: HIIT	25	− 0.62 (− 1.12 to − 0.12)	**0.01**	0% (0.54)
Duration: long term	22	− 1.10 (− 1.67 to − 0.53)	**0.0002**	0% (0.94)
Duration: short term	24	− 0.38 (− 1.22 to 0.46)	0.38	25% (0.13)
BMI: 18.5− 24.9 kg/m^2^	4	− 0.45 (− 2.87 to 1.97)	0.72	2% (0.38)
BMI: ≥ 25 kg/m^2^	37	− 0.74 (− 1.19 to − 0.30)	**0.001**	1% (0.46)
Mode: cycling	29	− 0.90 (− 1.43 to − 0.36)	**0.001**	0% (0.80)
Mode: run/walk/jog	14	− 0.66 (− 1.71 to 0.38)	0.21	36% (0.09)
HIIT volume: low	11	− 1.14 (− 1.94 to − 0.35)	**0.005**	0% (0.76)
HIIT volume: high	7	− 0.03 (− 0.99 to 0.92)	0.94	2% (0.41)
*Body mass (kg)*	51	0.40 (− 0.48 to 1.28)	0.37	0% (0.98)
Protocol: SIT	24	0.31 (− 1.49 to 2.10)	0.74	0% (0.92)
Protocol: HIIT	29	0.43 (− 0.58 to 1.43)	0.41	0% (0.88)
Duration: long term	19	0.23 (− 1.41 to 1.88)	0.78	0% (0.91)
Duration: short term	32	0.39 (− 0.66 to 1.43)	0.47	0% (0.92)
BMI: 18.5− 24.9 kg/m^2^	9	0.96 (− 0.49 to 2.42)	0.19	0% (086)
BMI: ≥ 25 kg/m^2^	35	− 0.07 (− 1.09, 1.23)	0.91	0% (0.98)
Mode: cycling	35	0.65 (− 0.67, 1.97)	0.33	0% (0.97)
Mode: run/walk/jog	13	0.45 (− 0.77, 1.67)	0.47	0% (0.78)
HIIT volume: low	15	1.17 (− 0.69, 3.04)	0.22	0% (0.65)
HIIT volume: high	12	0.36 (− 0.95 to 1.67)	0.59	0% (0.87)
*Body mass index (kg/m*^*2*^*)*	40	0.06 (− 0.13 to 0.26)	0.51	0% (0.68)
Protocol: SIT	17	0.17 (− 0.43 to 0.78)	0.57	0% (0.59)
Protocol: HIIT	24	0.05 (− 0.15 to 0.25)	0.61	0% (0.58)
Duration: long term	14	− 0.31 (− 0.91 to 0.28)	0.3	0% (0.67)
Duration: short term	26	0.11 (− 0.09 to 0.31)	0.28	0% (0.60)
BMI: 18.5− 24.9 kg/m^2^	10	0.13 (− 0.09 to 0.34)	0.24	0% (0.60)
BMI: ≥ 25 kg/m^2^	29	− 0.31 (− 0.74 to 0.12)	0.15	0% (0.66)
Mode: cycling	25	0.34 (− 0.14 to 0.81)	0.17	0% (0.95)
Mode: run/walk/jog	13	0.02 (− 0.25 to 0.29)	0.86	2% (0.43)
HIIT volume: low	12	0.45 (− 0.34 to 1.23	0.26	0% (0.97)
HIIT volume: high	10	0.09 (− 0.12 to 0.30)	0.41	0% (0.72)
*Waist circumference (cm)*	18	0.94 (− 0.59 to 2.47)	0.23	0% (0.84)
Protocol: SIT	4	2.22 (− 1.82 to 6.26)	0.28	15% (0.32)
Protocol: HIIT	15	0.58 (− 1.10 to 2.27)	0.23	0% (0.84)
Duration: long term	10	0.74 (− 1.52 to 3.00)	0.52	0% (0.78)
Duration: short term	9	1.10 (− 0.97 to 3.37)	0.3	0% (0.60)
Mode: cycling	11	1.42 (− 0.87 to 3.71)	0.22	0% (0.80)
Mode: run/walk/jog	6	0.48 (− 1.64 to 2.60)	0.66	0% (0.55)
HIIT volume: low	10	0.92 (− 1.26 to 3.10)	0.41	0% (0.79)
HIIT volume: high	5	0.45 (− 2.35 to 3.26)	0.75	0% (0.71)
*Waist− to− hip ratio*	8	0 (− 0.01 to 0.01)	0.7	0% (0.47)
Duration: long term	4	− 0.01 (− 0.04 to 0.02)	0.7	39% (0.18)
Duration: short term	4	0 (− 0.01 to 0.01)	0.52	0% (0.73)
Mode: cycling	4	− 0.02 (− 0.06 to 0.01)	0.2	0% (0.67)
Mode: run/walk/jog	4	0.00 (− 0.01 to 0.01)	0.47	2% (0.38)
*Lean mass (kg)*	13	0.55 (− 0.53 to 1.63)	0.32	0% (0.71)
Protocol: SIT	5	0.61 (− 1.01 to 2.23)	0.46	0% (0.65)
Protocol: HIIT	8	0.51 (− 0.94 to 1.95)	0.49	0% (0.49)
Duration: long term	5	1.96 (− 0.21 to 4.13)	0.08	0% (0.74)
Duration: short term	8	0.13 (− 1.11 to 1.38)	0.83	0% (0.68)
Mode: cycling	5	0.55 (− 0.86 to 1.96)	0.45	0% (0.98)
Mode: run/walk/jog	7	0.17 (− 2.63 to 2.98)	0.9	18% (0.29)
*Fat− free mass (kg)*	10	1.40 (− 0.19 to 3.00)	0.08	0% (0.78)
Protocol: SIT	4	0.78 (− 1.40 to 2.95)	0.48	0% (0.95)
Protocol: HIIT	6	2.13 (− 0.21 to 4.47)	0.07	0% (0.45)
*Fat mass (kg)*	23	− 0.26 (− 0.98 to 0.46)	0.48	0% (0.72)
Protocol: SIT	9	− 0.40 (− 1.95 to 1.16)	0.62	0% (0.82)
Protocol: HIIT	15	− 0.23 (− 1.06 to 0.61)	0.59	2% (0.43)
Duration: long term	10	− 0.72 (− 1.65 to 0.21)	0.13	0% (0.46)
Duration: short term	13	0.44 (− 0.70 to 1.59)	0.45	0% (0.90)
Mode: cycling	18	0.14 (− 0.72 to 1.29)	0.75	0% (0.98)
Mode: run/walk/jog	4	0.13 (− 1.54 to 1.80)	0.88	0% (0.57)
HIIT volume: low	6	0.53 (− 0.84 to 1.90)	0.45	0% (0.74)
HIIT volume: high	6	0.45 (− 0.97 to 1.879	0.54	0% (0.93)
*Visceral adipose tissue*^a^	9	− 0.08 (− 0.34, 0.17)	0.52	0% (0.71)
Duration: long term^a^	5	− 0.26 (− 0.58, 0.06)	0.11	0% (0.91)
Duration: short term^a^	4	0.21 (− 0.20, 0.63)	0.31	0% (0.53)
*Abdominal fat (total)*^a^	8	− 0.03 (− 0.27, 0.21)	0.8	0% (0.97)
Duration: long term^a^	4	− 0.18 (− 0.52, 0.17)	0.32	0% (1.00)
Duration: short term^a^	4	0.10 (− 0.23, 0.42)	0.56	0% (0.65)
*Abdominal fat (subcutaneous)*^a^	6	0.05 (− 0.25, 0.35)	0.73	0% (0.71)
*Abdominal fat (android)*^a^	9	− 0.06 (− 0.33, 0.20)	0.65	0% (0.89)
*Abdominal fat (gynoid)*^a^	8	− 0.65 (− 1.44, 0.14)	0.11	81% (< 0.00001)
Duration: long term^a^	4	− 1.36 (− 2.82, 0.11)	0.07	88% (< 0.00001)
Duration: short term^a^	4	− 0.03 (− 0.51, 0.44)	0.89	0% (0.71)

*BMI* body mass index, *HIIT* high-intensity interval training, *MICT* moderate-intensity continuous training, *SIT* sprint interval training

^a^Reported as standardized mean difference; bold text signifies statistically significant results

## Discussion

**Fig. 5:**
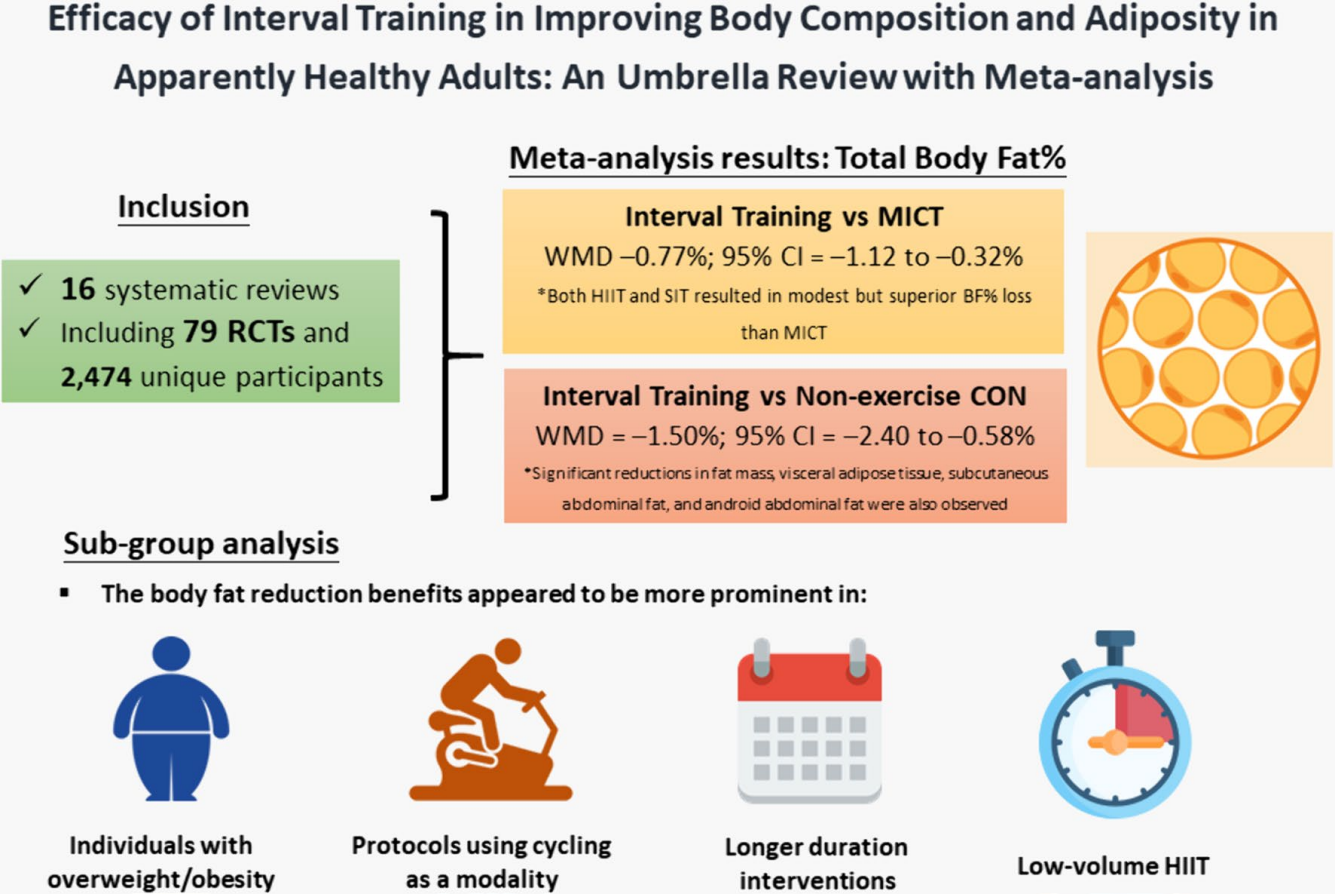
Graphical representation of the efficacy of interval training in reducing body adiposity in apparently healthy adults. *CI* confidence interval, *CON* control, *HIIT* high-intensity interval training, *MICT* moderate-intensity continuous training, *RCTs* randomized controlled trials, *SIT* sprint interval training, *WMD* weighted mean difference

To the best of our knowledge, this is the first umbrella review with large-scale meta-analysis examining the efficacy of interval training, including HIIT and SIT, in improving body composition and adiposity in adults. We identified 16 systematic reviews, reporting the findings of 79 original RCTs, involving 2474 unique participants. The findings of our umbrella review support the widespread efficacy of interval training in improving a range of body composition and adiposity-related outcomes, such as total BF%, FM, VAT, AF_subcutaneous_, and AF_android_ compared with CON. While the difference appeared modest, our analysis also revealed that both HIIT and SIT resulted in a superior reduction in BF% compared to MICT. This effect was particularly pronounced in individuals with overweight/obesity and in interventions with longer durations, as well as in protocols with cycling as the exercise modality and low HIIT volume (see Fig. 5 for the graphical representation of findings).

Several mechanisms that may contribute to the observed fat loss associated with interval training have been documented in the literature [104, 105]. One commonly proposed mechanism is the phenomenon known as excess postexercise oxygen consumption (EPOC). Interval training involves short bursts of intense exercise followed by brief recovery periods. This pattern creates an oxygen debt that the body needs to repay during the recovery period, leading to increased calorie burning and fat oxidation after exercise cessation [106]. The metabolic rate remains slightly elevated in response to exercise intensity, ranging from an hour to several hours with higher intensities [107, 108]. However, given that many interval training protocols involve a low volume of exercise, it is debatable whether EPOC can lead to a greater total energy deficit when compared with MICT, which tends to result in greater energy expenditure during the exercise bout [109]. Thus, hormonal changes induced by interval training may also play a role in fat loss. High-intensity exercise (i.e., above 65% maximal oxygen uptake [VO_2max_]) stimulates the release of growth hormone and catecholamines (epinephrine and norepinephrine), which elevate tissue lipolysis [110, 111]. Recent evidence suggests that interval training may be particularly effective in reducing adipose tissues in the visceral regions, as the significantly increased catecholamine responses during interval training favor lipolysis via beta-adrenergic receptors located in visceral adipose tissue [112]. Furthermore, exercise may trigger changes in the levels of circulating appetite-related hormones and metabolites, as well as sensations of hunger and satiety [113]. These responses also appear to be dependent on exercise intensity [114], as higher intensity exercise was found to promote appetite suppression [115]. Interval training has been shown to have a favorable impact on appetite-regulating hormones, such as leptin and ghrelin, leading to a decrease in postexercise appetite and potentially lower energy intake [105, 116]. Collectively, EPOC, enhanced catecholamine release that promotes tissue lipolysis, and decreased postexercise appetite provide a scientific basis for the potency of interval training for reducing adiposity.

Regarding the clinical significance of our results, it is acknowledged that there is currently no universally agreed-upon minimal clinically meaningful or cutoff value of BF% reduction in relation to cardiometabolic risk [117]. This value may vary depending on individual factors and the specific guideline being referenced. However, a recent epidemiological study suggested cutoff values of 25.8% for men and 37.1% for women for predicting the cardiovascular risk factors related to obesity [118]. Considering these benchmarks, we recognize that the observed WMD in BF% in our study may appear modest when comparing interval training with CON (− 1.5%; 95% CI − 2.41% to − 0.58%) and MICT (− 0.77%; 95% CI − 1.22% to − 0.32%). These differences are only incrementally higher than the typical biological error of laboratory-standard body composition techniques such as dual-energy X-ray absorptiometry [119]. The relatively small magnitude of improvement raises questions about the clinical significance of our results, despite their statistical significance. Nonetheless, our subgroup analysis revealed greater benefits in longer duration interventions (≥ 12 weeks) and in individuals with overweight/obesity, who are the priority target for public health promotion. Additionally, it is important to note that most included studies in our review controlled for participants’ diets to minimize the confounding effects of diet on body composition parameters. These findings indicate that the impact of interval training on BF% reduction may be amplified in individuals with a relatively high baseline BF% who adhere to an energy-restrictive diet, as typically prescribed for weight management, over a sustained period of engagement.

Another noteworthy finding from the subgroup analysis was that cycling appeared to be more efficacious than running/walking/jogging in reducing BF%. One possible explanation is that while all these modalities were commonly employed in our included studies, cycling is a nonweight-bearing activity that is gentler on the joints. This lower impact nature of cycling may make it a suitable exercise option, particularly for individuals with overweight/obesity or musculoskeletal issues, as it reduces stress on the joints and lowers the risk of injury [2, 120]. This may in turn enable individuals to sustain longer and more intense exercise sessions, leading to more efficient fat loss. Additionally, our subgroup analysis indicates that HIIT protocols with low volume (i.e., < 15 min of high-intensity exercise per session) yielded comparable effects for most body composition outcomes and possibly superior improvements in BF% reduction, as compared with interventions with high-volume protocols. Existing literature suggests that low-volume HIIT has the potential to rapidly enhance cardiometabolic adaptations, including increased mitochondrial biogenesis and improved insulin sensitivity, through enhanced molecular signalling activities [121, 122]. These adaptations are believed to contribute to an improved capacity for fat oxidation, which can enhance metabolic health and facilitate the reduction of body fat, particularly in individuals with metabolic disorders and impaired fatty acid oxidation [123]. However, from a physiological standpoint, the mechanisms proposed for the benefits of low-volume HIIT would also apply to high-volume HIIT. Moreover, high-volume HIIT has the added benefit of higher overall exercise session energy expenditure, which should theoretically lead to greater fat loss if all other factors are equal. The small actual differences observed, while modestly larger than technical/biological error, could also be due to uncontrolled or unaccounted for factors; although, a similar counterintuitive finding has been shown for reduced-volume SIT before [124]. Further research with stronger statistical power is needed to fully elucidate the precise mechanisms contributing to the observed effects of HIIT protocols with varying volumes on body composition outcomes. Another advantage of low-volume protocols is their perceived “time efficiency” [122], which may make it easier for individuals to incorporate them into their routine. However, it is worth noting that these time-saving benefits may not be substantial when considering factors such as warm-up/cool-down periods and rest intervals. Nevertheless, from a practical perspective, our results suggest that low-volume HIIT can serve as a viable exercise alternative or complement to more traditional forms of aerobic exercise regimen, such as high-volume HIIT and MICT, for improving body composition and adiposity.

There is an understandable concern about the practicality and safety of implementing interval training in less fit or previously inactive populations, including some individuals who with overweight/obesity. For instance, a recent commentary has raised doubts about the long-term sustainability of HIIT [125]. The transition from short-term supervised exercise programs to long-term self-directed interventions in research settings has been linked to decreased participation, partly due to the ongoing need for supervision, monitoring, and support. However, this concern does not seem unique to HIIT. A recent systematic review and meta-analysis conducted by Santos et al. [126], which included 188 unique studies with a total of 8928 participants, revealed that in unsupervised, real-world interval training interventions (inclusive of both HIIT and SIT), the average adherence rate (i.e., completion of unsupervised physical activity) was moderate at 63%, which was comparable with the adherence rate of MICT interventions at 68%. Furthermore, the analysis showed that compliance rates (i.e., supervised intervention attendance) to both interval training and MICT were high among insufficiently active adults and adults with a medical condition, with rates of 89% and 92%, respectively. These high compliance rates align with the modest discontinuation rate (~ 15%) reported in the included RCTs within our review that reported dropout rates specifically in interval training programs. Previous studies have demonstrated that interval training performed at high intensities appears to be safe, well tolerated, and achievable, even when applied in clinical populations with low initial fitness levels (e.g., patients with coronary artery disease, heart failure, and various forms of cancer) [14, 127–129]. Nevertheless, inactive individuals with cardiovascular risk factors should be encouraged to undergo a medical evaluation before initiating any exercise program [2]. Although current research suggests that interval training is safe for most healthy individuals, it is prudent for fitness and health professionals to perform proper prescreening and deliver all exercise programming in a progressive manner with adequate supervision.

A limitation of this umbrella review is that most of the included systematic reviews were rated as critically low (*n* = 6) or low (*n* = 6), based on the AMSTAR-2 quality rating. Specifically, only a small number of reviews referred to a predefined methodology or assessed the impact of RoB on the results. None of the studies provided a list of excluded studies with reasons for exclusions or reported on the sources of funding for the included studies. This underscores the importance of exercising caution when interpreting certain included reviews and highlights the need for well-conducted systematic reviews in this particular field. Nonetheless, our methodological quality assessment of all 78 included RCTs indicated relatively high PEDro scores, with most RCTs rated as excellent (41%) or good (33%). This suggests that our meta-analysis is expected to contribute to a strong and reliable evidence base on interval training and its effects on body composition and adiposity. Furthermore, it is noted that the terms HIIT and SIT were defined somewhat inconsistently across studies. For instance, Bartlett et al. [29] initially described their protocols as HIIT, involving repeated high-intensity sprints lasting between 15 and 60 s at an intensity exceeding 90% HRmax. However, considering the recognized time delay in achieving a steady-state HR (typically exceeding 1 min), any protocol utilizing short (e.g., ≤ 1-min) intervals and relying solely on HR% should be subjected to scrutiny when distinguishing between SIT and HIIT. Lastly, it should be noted that the target population of this umbrella review and meta-analysis was apparently healthy adults without acute or chronic diseases. Therefore, caution should be taken when generalizing the results to other populations, such as children and adolescents, as well as different clinical populations (e.g., persons with type 2 diabetes, metabolic syndrome, or hypertension).

## Conclusions

This novel umbrella review with large-scale meta-analysis provides robust evidence supporting the efficacy of interval training, including both HIIT and SIT, in reducing adiposity in adults. Interval training demonstrated significant but modestly greater reductions in total BF% compared with traditional MICT and nonactive control groups. These benefits appeared to be more prominent in individuals with overweight/obesity and longer duration interventions (≥ 12 weeks), as well as in protocols employing cycling as a modality and using low-volume HIIT (i.e., < 15 min of high-intensity exercise per session). Our findings can help address the existing limitations in PA guidelines regarding the recommendation of interval training as a viable exercise strategy for improving body composition and adiposity. Further research and implementation efforts are warranted to optimize the integration of interval training into comprehensive obesity prevention and management programs and to evaluate the impact of different interval training interventions on obesity-related comorbidities.

## Supplementary Information

Below is the link to the electronic supplementary material.

Supplementary file1 (DOCX 14 KB)

Supplementary file2 (DOCX 34 KB)

Supplementary file3 (DOCX 31 KB)

Supplementary file4 (DOCX 31 KB)

## Declarations

### Availability of data and material

The datasets analyzed in this review are available from the corresponding author on reasonable request.
