# Supplementary material for: Efficacy of Interval Training in Improving Body Composition and Adiposity in Apparently Healthy Adults: An Umbrella Review with Meta-Analysis
Source: Sports Med. 2024 Jul 14;54(11):2817–40. doi: 10.1007/s40279-024-02070-9 (PMC11560999; doi:10.1007/s40279-024-02070-9)
Supplement: Supplementary file 1 — Supplementary file1 (DOCX 14 KB) [file 40279_2024_2070_MOESM1_ESM.docx]

**Supplement Table S1** Search strategy

| **Terms** | **Search Strategy** |
| --- | --- |
| High intensity interval training | (“high-intensity interval training” OR “high-intensity interval exercise*” OR “high-intensity intermittent exercise” OR “high-intensity intermittent training” OR “sprint interval training” OR “sprint interval exercise” OR “interval training” OR “interval exercise” OR “HIIT” OR "high intensity training" OR "high intensity exercise*" OR "high intensity activit*" OR “vigorous training” OR "vigorous intensity exercise*" OR "vigorous activit*" OR “aerobic interval training” OR “aerobic interval exercise”) |
| Body adiposity | (“weight loss” OR “fat loss” OR “lean loss” OR “lean body loss” OR “visceral fat” OR “abdominal fat” OR “fat mass” OR “body composition” OR “weight maintenance” OR “weight regain” OR “adiposity" OR “waist circumference”) |
| Reviews | (“systematic review” OR “systematic literature review” OR “systematic” OR “review” OR “cochrane database syst rev” OR “meta-analysis” OR “metaanalysis” OR “meta analysis” OR “meta-analytic review” OR “meta analy*” OR “meta-analy*” OR “metaanaly*” OR “metareview” OR “meta-review”) |
