# Supplementary material for: Efficacy of Interval Training in Improving Body Composition and Adiposity in Apparently Healthy Adults: An Umbrella Review with Meta-Analysis
Source: Sports Med. 2024 Jul 14;54(11):2817–40. doi: 10.1007/s40279-024-02070-9 (PMC11560999; doi:10.1007/s40279-024-02070-9)
Supplement: Supplementary file 2 — Supplementary file2 (DOCX 34 KB) [file 40279_2024_2070_MOESM2_ESM.docx]

**Supplementary Table S2** Intervention characteristics of included RCTs

| **Studies** |  | | **Intervention** | | | | | | | |
| --- | --- | --- | --- | --- | --- | --- | --- | --- | --- | --- |
| **Author, Year** | **Characteristic** | **Sample Size*** | **Duration (wks)** | **Frequency (times/wk)** | **Time (min)** | **Intensity** | **Mode** | **Set / bouts** | **Work:Rest (sec)** | **Recovery** |
| Abderrahman et al 2013 | Physical education students | 24 | 7 | 3 | NR | 105% Maximal aerobic velocity | Running | SITa: 2x8-10 SITb: 2x12-15 | 30 : 30 | HIITa: Active (50% MAV) HIITb: Passive |
| Ahmadizad et al 2015 | Overweight | 30 | 6 | 3 | 30-70 | 90% VO2max | Running | 8 | 60 : 120 | Active |
| Arad et al 2015 | Healthy, Premenopausal, Overweight/obese | 20 | 14 | 3 | 24 | 75-90% HRR | Cycling | 4 | 30-60 : 180-210 | Active (50% HRR) |
| Astorino et al 2013 | Healthy, sedentary | 27 | 12 | 3 | 12.25 - 21.25 | 85-100% HRmax | Cycling | 6-10 | 60 : 75 | Active (40W) |
| Ballin et al. 2019 | Central obesity | 72 | 10 | 3 | 18-36 | BorgCR10 scale: 6-7 | Circuit-based dynamic body-weight exercise | NR | 40 : 20 | NR |
| Bartlett et al 2017 | Healthy | 27 | 10 | 3 | 18-25 | >90% HRmax | Cycling | NR | 15-60 : 45-120 | Active |
| Bouri et al 2015 | Overweight | 14 | 8 | NR | 33 | 85-95 HRmax | Walking/Running | 4 | 240 : 180 | Active (50-70 HRmax) |
| Burgomaster et al 2008 | Healthy | 20 | 6 | 3 | 15.5-25.5 | All-out | Cycling | 4-6 | 30 : 270 | Active |
| Cheema et al 2015 | Overweight/Obese | 12 | 12 | 4 | 50 | 86-89 HRmax | Boxing drills | 3 | 120 : 60 | Passive |
| Cocks et al 2013 | Sedentary | 16 | 6 | 3 | 15.5-25.5 | All-out | Cycling | 4-6 | 30 : 300 | Active |
| Cocks et al 2016 | Obese | 16 | 4 | 3 | 10-17.5 | 200% Wmax | Cycling | 4-7 | 30 : 120 | active |
| Cooper et al 2016 | Healthy | 59 | 12 | 3 | 21-42 | All-out | Cycling | 4-10 | 30 : 180 | A-SIT: Active P-SIT: Passive |
| Dunham and Harms 2012 | Healthy | 15 | 4 | 3 | 23 | 90% VO2max | Cycling | 5 | 60 : 180 | Active (20W) |
| Edge et al 2006 | Students | 16 | 5 | 3 | 12-29 | 120-140% Lactate threshold | Cycle | 2-10 | 120 : 60 | Passive |
| Eimarieskandari et al 2012 | Obese | 20 | 8 | 3 | 33 | 85-95% HRmax | Walking / Jogging | 4 | 240 : 180 | Active (50-70 HRmax) |
| Elmer et al 2016 | Healthy | 12 | 8 | 3 | >30 | 90-110 VO2max | Running | 12 | 60 : 60 | Active (50% VO2max) |
| Eskelinen et al 2015 | Healthy | 26 | 2 | 3 | 14-23 | Maximal | Cycling | 4-6 | 30 : 240 | Passive/ Active |
| Fedewa et al 2018 | Overweight/ Obese | 44 | 6 | 3 | 14.5-27.5 | Maximal | Cycling | 5-7 | 30 : 240 | Active |
| Gahreman et al 2016 | Healthy | 48 | 12 | 3 | 30 | 85-90 HRmax | Cycling | 60 | 8 : 12 | Active |
| García-Pinillos et al 2019 | Active | 90 | 12 | 3 | 35-50 | NR | Circuit strength training, Walking & Running | NR | 20 : 40 / 40 :20 | NR |
| Gerosa-Neto et al 2019 | Obese | 32 | 6 | 3 | 29 | 100% VO2max | Running | 10 | 60 : 60 | Passive |
| Gillen et al 2016 | Sedentary | 25 | 12 | 3 | 12 | All out | Cycling | 3 | 20 :120 | Acctive |
| Gormley et al 2008 | NR | 55 | 6 | 3 | 30-45 | 90-100% HRR | Cycle | 5 | 300 : 300 | Active (50% HRR) |
| Gripp et al 2021 | Police officier | 22 | 8 | 3 | 14.8-19.9 | 85-100% V-shuttle max | Shuttle run | 7-10 | 20m : 1 min | Passive |
| Helgerud et al 2007 | Healthy | 40 | 8 | 3 | 15/15 HIIT: 36.25 4x4HIIT: 38 | 90-95% HRmax | Running | 15/15 HIIT: 47 4x4HIIT: 4 | 15/15 HIIT 15 : 15 4x4HIIT 240 : 180 | Active (70% HRmax) |
| Heydari & Boutcher 2012 | Overweight | 46 | 12 | 3 | 20 | 80-90% HRmax | Cycling | 60 | 8 : 12 | Passive |
| Heydari & Boutcher 2013 | Overweight | 46 (same sample) | 12 | 3 | 20 | 80-90% HRmax | Cycling | 60 | 8 : 12 | Passive |
| Heydari et al 2013a | Overweight/ Obese | 34 | 12 | 3 | 20 | 80-90% HRmax | Cycling | 60 | 8 : 12 | Passive |
| Heydari et al 2013b | Inactive | 38 | 12 | 3 | 20 | 80-90% HRmax | Cycling | 60 | 8 : 12 | Passive |
| Higgins et al 2016 | Overweight/ obese | 52 | 6 | 3 | 18.5-31.5 | All out | Cycle | 5-7 | 30 : 240 | active |
| Hornbuckle et al 2018 | Overweight/ obese | 14 | 16 | 3 | 38 | 80-90% HRmax | Running | 7 | 60 : 180 | Active (60-70% HRmax) |
| Hwang et al 2016 | Healthy | 43 | 8 | 4 | 40 | 90% HRmax | All-extremity air-baked ergometer | 4 | 240 : 180 | Active (70% HRmax) |
| Jabbour et al 2017 | Obese | 24 | 6 | 3 | 15 | Maximal | Cycling | 6 | 6 : 120 | Passive |
| Jiménez-García et al 2019 | NR | 73 | 12 | 2 | 25 | 90-96% HRmax | TRX | NR | 240 : 180 | Active (50-70HRmax) |
| Keating et al 2014 | Overweight | 38 | 12 | 3 | 20-24 | 120% VO2peak | Cycle | 4-6 | 30-60 : 120-180 | active |
| Kong et al 2016a | Obese | 26 | 5 | 4 | 20 | All out | Cycling | 60 | 8 : 12 | Passive |
| Kong et al 2016b | Overweight / obese | 18 | 5 | 4 | 20 | All out | Cycling | 60 | 8 : 12 | Passive |
| Lunt et al 2014 | Overweight/ obese | 32 | 12 | 3 | SIT: 24.5-40 HIIIT: 40 | SIT: All-out HIIT: 85-95% HRmax | Cycling | 4 | 4:3 | Active |
| MacPherson et al 2011 | Healthy | 20 | 6 | 3 | 45 | Maximal | Running | 4-6 | 30 : 240 | Active/ Passive |
| Mader et al 2001 | Overweight | 14 | 10 | 3 | 50 | 80% VO2max | Cycling | 25 | 40 : 80 | Active (35% VO2max) |
| Malin et al 2020 | Obesity | 26 | 2 (13 days) | 7 (12 sessions) | 30-45 | 90% HRmax | Cycling | NR | 180 : 180 | Active (50% HRmax) |
| Matsuo et al 2014a | Sedentary | 24 | 8 | 3 | 18 | 85% VO2max | Cycling | 5 | 180 : 120 | Active (50% VO2max) |
| Mirghani and Yousefi 2015 | Overweight/ obesity | 24 | 4 | 3 |  | 80% HRR | Running | 4-10 | HIIT 60/60 60 : 60 HIIT 60/30 60 : 30 | Passive |
| Moreira et al 2008 | Overweight, sedentary | 16 | 12 | 3 | 20-60 | 20% above anaerobic threshold | Cycling | NR | 2:1 | NR |
| Musa et al 2009 | Untrained | 36 | 8 | 3 | 40 | 90% HRmax | Run | 4 | 1:1 | NR |
| Nalcakan 2014 | Healthy, recreationally active | 15 | 7 | 3 | 30 | All-out | Cycling | 4-6 | 30: 270 | Passive |
| Nemoto et al 2007 | Healthy | 139 | 4 | 4-5 | NR | 70-85%VO2max | Walking | 5 | 180 : 120/180 | Active (40% VO2max) |
| Nie et al 2018 | Obese | 43 | 12 | 3-4 | NR (till 300 kJ) | 90% VO2max | Cycling | NR (till 300 kJ) | 240 : 180 | Passive |
| Nybo et al 2010 | Inactive | 36 | 12 | 2 | 20 | >95% HRmax | Running | 5 | 120 : 60 | NR |
| Panissa et al 2016 |  | 23 | 6 | 3 | 22 | 90% HRmax | Cycling | 15 | 60 : 30 | Active (60% HRmax) |
| Poon et al 2020 | Overweight/ obese | 24 | 8 | 3 | 20 | 80–90% HRmax | Running | 10 | 60 : 60 | Active (50% HRmax) |
| Poon et al 2021 | Overweight/ obese | 42 | 16 | 3 | 24 | 80–90% HRmax | Running | 12 | 60 : 60 | Active (50% HRmax) |
| Rakobowchuk et al 2008 | Healthy | 20 | 6 | 3 | 17-28 | Maximal sprints | Cycling | 4-6 | 30 : 300 | Passive |
| Ramirez-Velez 2017 | Healthy | 20 | 12 | 3 | 35-55 | 85-95% HRR | Running | 4 | 240 : 240 | Active (65% HRR) |
| Rebold 2013 |  | 25 | 8 | 2 | 20 | All-out | Aquatic treadmill running | 8 | 20 : 10 | Passive |
| Relijc et al 2018 | Sedentary | 27 | 8 | 2 | HIIT 2x4: 15 HIIT 5x1: 14 | 85-95% HRmax | Cycling | HIIT 2x4: 8 HIIIT 5x1: 5 | HIIT 2x4: 240 : 120 HIIT 5x1: 60 : 60 | Active |
| Rowley et al 2017 | Overweight/ obese | 12 | 12 | 3 | 14-41 | Maximal | Running | 4-10 | 30 : 240 | Active |
| Sandvei et al 2012 | Healthy, sedentary to moderately trained | 23 | 8 | 3 | 14.5-38 | Maximal | Running | 5-10 | 30 : 180 | Passive |
| Sasaki et al 2014 | Sedentary | 24 | 4 | 3 | 15 | 85% VO2max | Cycling | 10 | 60 : 30 | Passive |
| Sawyer et al 2016 | Obese | 18 | 8 | 3 | 29 | 90-95% HRmax | Cycling | 10 | 60 : 60 | Active |
| Schubert et al 2017 | Healthy | 30 | 4 | 3 | SIT 10-15 HIIT 16-20 | SIT All out HIIT 90% Peak power output | Cycling | SIT 3-5 HIIT 6-8 | SIT 20 : 120 HIIT 60 : 60 | Active (10% Peak power output) |
| Sculthorpe et al 2017 | Sednetary | 33 | 6 | 5 | 18 | 50% Peak power | Cycling | 6 | 30 : 180 | Active |
| Shepherd et al 2013 | Healthy | 16 | 6 | 3 | 17-28 | All out | Cycling | 4-6 | 30 : 300 | Active |
| Shepherd et al 2015 | Inactive | 78 | 10 | 3 | 18-25 | >90% HRmax | Cycling | 4-12 | 15-60 : 45-120 | Active |
| Sijie et al 2012 | Overweight | 52 | 12 | 5 | 42 | 85% VO2max | Running | 5 | 180 : 180 | Active (50% VO2max) |
| Sim et al 2015 | Overweight | 30 | 12 | 3 | 30-45 | 120% VO2peak | Cycle | NR | 15 : 60 | Active (32% VO2max) |
| Skleryk et al 2013 | Obese | 16 | 2 | 3 | 11-17 | Maximal | Cycling | 8-12 | 10 : 80 | Passive |
| Smith-Ryan et al 2016 | Overweight | 30 | 3 | 3 | HIIT 1min 19 HIIT 2min 14 | HIIT 1min 90% VO2max HIIT 2min 80-100% VO2max | Cycling | HIIT 1min 10 HIIT 2min 5 | HIIT 1min 60 : 60 HIIT 2min 120 : 60 | Active |
| Tong et al 2018 | Obese | 46 | 12 | 3-4 | SIT: 8 HIIT: 27.6-48.2 | SIT: All out HIIT: 90% VO2 peak | Cycling | SIT: 80 HIIT: till 200-400 kJ | SIT: 6 : 9 HIIT: 240 : 180 | Passive |
| Trapp et al 2008 | Healthy, inactive | 45 | 15 | 3 | 5-20 | Maximal | Cycling | 60 | 8 : 12 | Active |
| Tsekouras et al 2008 | Healthy, sedentary | 15 | 8 | 3 | 32 | 90% VO2max | Running | 4 | 240 : 240 | Active (60% VO2max) |
| Umamaheswari et al 2017 | Overweight | 72 | 15 | 3 | 20 | 75-84% HRR | Cycling | 60 | 8 : 12 | Active |
| Vella et al 2017 | Overweight/ Obese | 17 | 8 | 3-4 | 30 | 75-80% HRR | Running/ Cycling/ Elliptical | 10 | 60 : 60 | Active (35-40 HRR) |
| Wallman et al 2009 | Overweight/ Obese | 21 | 8 | 4 | 30 | 90-105%VO2peak | Cycling | 10 | 60 : 120 | Active (30-45% VO2max) |
| Zhang et al 2015 | Overweight/ Obese | 35 | 12 | 4 | 40 | 85-95 HRmax | Running | 4 | 240 : 180 | Active (50-60% HRmax) |
| Zhang et al 2017 | Overweight/ Obese | 43 | 12 | 3-4 | Till 200-300 kJ | 90% VO2max | Cycling | Till 200-300 kJ | 240 : 180 | Passive |
| Zhang et al 2021 | Obesity | 59 | 12 | 3-4 | Till 200 kJ | SIT all-out All-out ST-120 120% VO2 max HIIT 90% VO2 peak | Cycling | Till 200 kJ | SIT all-out 6 : 9 SIT-120 60 : 90 HIIT 240 : 180 | Passive |

*included for data analysis
