# Supplementary material for: Efficacy of Interval Training in Improving Body Composition and Adiposity in Apparently Healthy Adults: An Umbrella Review with Meta-Analysis
Source: Sports Med. 2024 Jul 14;54(11):2817–40. doi: 10.1007/s40279-024-02070-9 (PMC11560999; doi:10.1007/s40279-024-02070-9)
Supplement: Supplementary file 3 — Supplementary file3 (DOCX 31 KB) [file 40279_2024_2070_MOESM3_ESM.docx]

**Supplementary Table S3** Modified PEDro ratings (8-point) of included RCTs

| **Study** | **1** | **2** | **3** | **4** | **5** | **6** | **7** | **8** | **Total** | **Rating** |
| --- | --- | --- | --- | --- | --- | --- | --- | --- | --- | --- |
| Abderrahman et al 2013 | 1 | 1 | 0 | 1 | 1 | 1 | 1 | 1 | 6 | Excellent |
| Ahmadizad et al 2015 | 1 | 1 | 0 | 1 | 1 | 1 | 1 | 1 | 6 | Excellent |
| Arad et al 2015 | 1 | 1 | 0 | 1 | 0 | 0 | 1 | 1 | 4 | Fair |
| Astorino et al 2013 | 1 | 1 | 0 | 1 | 1 | 0 | 1 | 1 | 5 | Good |
| Ballin et al. 2019 | 1 | 1 | 1 | 1 | 1 | 0 | 1 | 1 | 6 | Excellent |
| Bartlett et al 2017 | 1 | 1 | 0 | 1 | 0 | 0 | 1 | 1 | 4 | Fair |
| Bouri et al 2015 | 1 | 1 | 0 | 1 | 1 | 1 | 1 | 1 | 6 | Excellent |
| Burgomaster et al 2008 | 1 | 1 | 0 | 1 | 1 | 1 | 1 | 1 | 6 | Excellent |
| Cheema et al 2015 | 1 | 1 | 1 | 1 | 0 | 1 | 1 | 1 | 6 | Excellent |
| Cocks et al 2013 | 1 | 1 | 0 | 1 | 1 | 1 | 1 | 1 | 6 | Excellent |
| Cocks et al 2016 | 1 | 1 | 0 | 1 | 1 | 0 | 1 | 1 | 5 | Good |
| Cooper et al 2016 | 1 | 1 | 0 | 1 | 1 | 0 | 1 | 1 | 5 | Good |
| Dunham and Harms 2012 | 0 | 1 | 0 | 1 | 0 | 0 | 1 | 1 | 4 | Fair |
| Edge et al 2006 | 1 | 1 | 0 | 1 | 1 | 0 | 1 | 1 | 5 | Good |
| Eimarieskandari et al 2012 | 1 | 1 | 0 | 1 | 1 | 1 | 1 | 1 | 6 | Excellent |
| Elmer et al 2016 | 1 | 1 | 0 | 1 | 1 | 1 | 1 | 1 | 6 | Excellent |
| Eskelinen et al 2015 | 1 | 1 | 0 | 1 | 1 | 0 | 1 | 1 | 5 | Good |
| Fedewa et al 2018 | 1 | 1 | 0 | 1 | 0 | 0 | 1 | 1 | 4 | Fair |
| Gahreman et al 2016 | 1 | 1 | 0 | 1 | 0 | 1 | 1 | 1 | 5 | Good |
| García-Pinillos et al 2019 | 1 | 1 | 0 | 1 | 1 | 0 | 1 | 1 | 5 | Good |
| Gerosa-Neto et al 2019 | 1 | 1 | 0 | 1 | 0 | 0 | 1 | 1 | 4 | Fair |
| Gillen et al 2016 | 0 | 1 | 0 | 1 | 1 | 1 | 1 | 1 | 6 | Excellent |
| Gormley et al 2008 | 1 | 1 | 0 | 1 | 1 | 0 | 1 | 1 | 5 | Good |
| Gripp et al 2021 | 1 | 1 | 0 | 1 | 0 | 1 | 1 | 1 | 5 | Good |
| Helgerud et al 2007 | 1 | 1 | 0 | 1 | 0 | 0 | 1 | 1 | 4 | Fair |
| Heydari & Boutcher 2012 | 1 | 1 | 0 | 1 | 0 | 0 | 1 | 1 | 4 | Fair |
| Heydari & Boutcher 2013 | 1 | 1 | 0 | 1 | 0 | 0 | 1 | 1 | 4 | Fair |
| Heydari et al 2013a | 1 | 1 | 0 | 1 | 0 | 0 | 1 | 1 | 4 | Fair |
| Heydari et al 2013b | 1 | 1 | 1 | 1 | 0 | 0 | 1 | 1 | 5 | Good |
| Higgins et al 2016 | 1 | 1 | 0 | 1 | 1 | 0 | 1 | 1 | 5 | Good |
| Hornbuckle et al 2018 | 1 | 1 | 0 | 1 | 0 | 1 | 1 | 1 | 5 | Good |
| Hwang et al 2016 | 1 | 1 | 0 | 1 | 0 | 1 | 1 | 1 | 5 | Good |
| Jabbour et al 2017 | 1 | 1 | 0 | 1 | 1 | 1 | 1 | 1 | 6 | Excellent |
| Jiménez-García et al 2019 | 1 | 1 | 1 | 1 | 1 | 0 | 1 | 1 | 6 | Excellent |
| Keating et al 2014 | 1 | 1 | 0 | 1 | 1 | 1 | 1 | 1 | 6 | Excellent |
| Kong et al 2016a | 1 | 1 | 0 | 1 | 0 | 0 | 1 | 1 | 4 | Fair |
| Kong et al 2016b | 1 | 1 | 0 | 1 | 0 | 0 | 1 | 1 | 4 | Fair |
| Lunt et al 2014 | 1 | 1 | 1 | 1 | 0 | 1 | 1 | 1 | 6 | Excellent |
| MacPherson et al 2011 | 0 | 1 | 0 | 1 | 1 | 1 | 1 | 1 | 6 | Excellent |
| Mader et al 2001 | 0 | 1 | 0 | 1 | 1 | 1 | 1 | 1 | 6 | Excellent |
| Malin et al 2020 | 0 | 1 | 0 | 1 | 1 | 1 | 1 | 1 | 6 | Excellent |
| Matsuo et al 2014a | 1 | 1 | 0 | 1 | 1 | 1 | 1 | 1 | 6 | Excellent |
| Mirghani and Yousefi 2015 | 1 | 1 | 0 | 1 | 1 | 1 | 1 | 1 | 6 | Excellent |
| Moreira et al 2008 | 1 | 1 | 0 | 1 | 0 | 1 | 1 | 1 | 5 | Good |
| Musa et al 2009 | 1 | 1 | 0 | 1 | 0 | 0 | 1 | 1 | 4 | Fair |
| Nalcakan 2014 | 0 | 1 | 0 | 1 | 1 | 1 | 1 | 1 | 6 | Excellent |
| Nemoto et al 2007 | 1 | 1 | 0 | 1 | 0 | 0 | 1 | 1 | 4 | Fair |
| Nie et al 2018 | 1 | 1 | 0 | 1 | 1 | 0 | 1 | 1 | 5 | Good |
| Nybo et al 2010 | 1 | 1 | 0 | 0 | 1 | 1 | 1 | 1 | 5 | Good |
| Panissa et al 2016 | 1 | 1 | 0 | 1 | 1 | 1 | 1 | 1 | 6 | Excellent |
| Poon et al 2020 | 1 | 1 | 1 | 1 | 1 | 1 | 1 | 1 | 7 | Excellent |
| Poon et al 2021 | 1 | 1 | 1 | 1 | 1 | 0 | 1 | 1 | 6 | Excellent |
| Rakobowchuk et al 2008 | 1 | 1 | 0 | 1 | 1 | 0 | 1 | 1 | 5 | Good |
| Ram et al 2020 | 1 | 1 | 0 | 1 | 0 | 0 | 1 | 1 | 4 | Fair |
| Ramirez-Velez 2017 | 1 | 1 | 1 | 1 | 1 | 1 | 1 | 1 | 7 | Excellent |
| Rebold 2013 | 1 | 1 | 0 | 1 | 1 | 1 | 1 | 1 | 6 | Excellent |
| Relijc et al 2018 | 1 | 1 | 0 | 1 | 0 | 1 | 1 | 1 | 5 | Good |
| Rowley et al 2017 | 1 | 1 | 0 | 1 | 0 | 0 | 1 | 1 | 4 | Fair |
| Sandvei et al 2012 | 1 | 1 | 0 | 1 | 1 | 0 | 1 | 1 | 5 | Good |
| Sasaki et al 2014 | 0 | 1 | 0 | 1 | 1 | 1 | 1 | 1 | 6 | Excellent |
| Sawyer et al 2016 | 1 | 1 | 0 | 1 | 0 | 0 | 1 | 1 | 4 | Fair |
| Schubert et al 2017 | 1 | 1 | 0 | 1 | 1 | 0 | 1 | 1 | 5 | Good |
| Schjerve et al 2008 | 1 | 1 | 0 | 1 | 1 | 1 | 1 | 1 | 6 | Excellent |
| Sculthorpe et al 2017 | 1 | 1 | 0 | 1 | 1 | 1 | 1 | 1 | 6 | Excellent |
| Shepherd et al 2013 | 0 | 1 | 0 | 1 | 1 | 1 | 1 | 1 | 6 | Excellent |
| Shepherd et al 2015 | 1 | 1 | 0 | 1 | 1 | 1 | 1 | 1 | 6 | Excellent |
| Sijie et al 2012 | 1 | 1 | 0 | 1 | 0 | 1 | 1 | 1 | 5 | Good |
| Sim et al 2015 | 1 | 1 | 0 | 1 | 1 | 1 | 1 | 1 | 6 | Excellent |
| Skleryk et al 2013 | 1 | 1 | 0 | 1 | 0 | 0 | 1 | 1 | 4 | Fair |
| Smith-Ryan et al 2016 | 1 | 1 | 0 | 1 | 1 | 0 | 1 | 1 | 5 | Good |
| Tong et al 2018 | 1 | 1 | 0 | 1 | 1 | 0 | 1 | 1 | 5 | Good |
| Trapp et al 2008 | 1 | 1 | 0 | 1 | 0 | 1 | 1 | 1 | 5 | Good |
| Tsekouras et al 2008 | 0 | 1 | 0 | 1 | 1 | 0 | 1 | 1 | 5 | Good |
| Umamaheswari et al 2017 | 1 | 1 | 0 | 1 | 0 | 0 | 1 | 1 | 4 | Fair |
| Vella et al 2017 | 1 | 1 | 0 | 1 | 1 | 0 | 1 | 1 | 5 | Good |
| Wallman et al 2009 | 1 | 1 | 0 | 1 | 1 | 1 | 1 | 1 | 6 | Excellent |
| Zhang et al 2015 | 1 | 1 | 0 | 1 | 0 | 0 | 1 | 1 | 4 | Fair |
| Zhang et al 2017 | 1 | 1 | 0 | 1 | 1 | 1 | 1 | 1 | 6 | Excellent |
| Zhang et al 2021 | 1 | 1 | 0 | 1 | 0 | 0 | 1 | 1 | 4 | Fair |

**Items:**

1. Eligibility criteria were specified (not included in score).

2. Subjects were randomly allocated to groups.

3. Allocation was concealed.

4. The groups were similar at baseline regarding the most important prognostic indicator.

5. Measures of at least one key outcome were obtained from more than 85% of the subjects initially allocated to groups.

6. All subjects for whom outcome measures were available received the treatment or control condition as allocated or, where this was not the case, data for at least one key outcome was analysed by intention to treat.

7. The results of between-group statistical comparisons are reported for at least one key outcome.

8. The study provides both point measures and measures of variability for at least one key outcome
