# Supplementary material for: Efficacy of Interval Training in Improving Body Composition and Adiposity in Apparently Healthy Adults: An Umbrella Review with Meta-Analysis
Source: Sports Med. 2024 Jul 14;54(11):2817–40. doi: 10.1007/s40279-024-02070-9 (PMC11560999; doi:10.1007/s40279-024-02070-9)
Supplement: Supplementary file 4 — Supplementary file4 (DOCX 31 KB) [file 40279_2024_2070_MOESM4_ESM.docx]

**Supplementary Table S4** List of excluded studies

| SR | RCT | Exclueded Reason Category | Reason |
| --- | --- | --- | --- |
| Wu | Chrois et al 2020 | Comparison | No comparsion group |
| Andreato | Larsen et al 2015 | Comparison | No comparsion group |
| Wu | Lepretre et al 2009 | Comparison | No comparsion group |
| Wu | Molmen et al 2012 | Comparison | No comparsion group |
| Wu | Sogaard et al 2018 | Comparison | No comparsion group |
| Batacan | Tjonna et al 2013 | Comparison | No comparsion group |
| Andreato | Whyte et al 2010 | Comparison | No comparsion group |
| Andreato | Boyd et al 2013 | Comparison | Not MICT or Control group |
| Wu | Bruseghini et al 2015 | Comparison | Not MICT or Control group |
| Wu | Moro et al 2017 | Comparison | Not MICT or Control group |
| Wang b | Nunes et al 2019 | Comparison | Not MICT or Control group |
| Guo | Sun et al 2019 | Comparison | Not MICT or Control group |
| Wu | Buckinx et al 2018 | Intervention | Both groups have HIIT |
| Wu | Buckinx et al 2019a | Intervention | Both groups have HIIT |
| Wu | Buckinx et al 2019b | Intervention | Both groups have HIIT |
| Guo | Gillen et al 2013 | Intervention | Both groups have HIIT |
| Batacan | Kendall et al 2009 | Intervention | Both groups have HIIT |
| Andreato | Mora-Rodriguez et al 2016 | Intervention | Both groups have HIIT |
| Wu | Morikawa et al 2018 | Intervention | Both groups have HIIT |
| Andreato | Tong et al 2011 | Intervention | Both groups have HIIT |
| Alzar-Teruel | Villanueva et al 2014 | Intervention | Both groups have HIIT |
| Batacan | Walter et al 2010 | Intervention | Both groups have HIIT |
| Wang b | Abd et al 2019 | Intervention | Not include HIIT |
| Wang b | Akbarpour et al 2013 | Intervention | Not include HIIT |
| Wang b | Auerbach et al 2013 | Intervention | Not include HIIT |
| Chang | Brennan et al 2019 | Intervention | Not include HIIT |
| Wang b | Brunelli et al 2015 | Intervention | Not include HIIT |
| Wang b | Chagas et al 2017 | Intervention | Not include HIIT |
| Wang b | Chow et al 2021 | Intervention | Not include HIIT |
| Chang | Cuff et al 2003 | Intervention | Not include HIIT |
| Chang | DiPietro et al 1998 | Intervention | Not include HIIT |
| Chang | Herzig et al 2014 | Intervention | Not include HIIT |
| Wang b | Ho et al 2013 | Intervention | Not include HIIT |
| Chang | Hong et al 2014 | Intervention | Not include HIIT |
| Chang | Irving et al 2008 | Intervention | Not include HIIT |
| Chang | Irwin et al 2003 | Intervention | Not include HIIT |
| Chang | Jung et al 2012 | Intervention | Not include HIIT |
| Wang b | Kang et al 2020 | Intervention | Not include HIIT |
| Chang | Keating et al 2015 | Intervention | Not include HIIT |
| Chang | Keating et al 2017 | Intervention | Not include HIIT |
| Wang b | Kelly et al 2004 | Intervention | Not include HIIT |
| Wang b | Kelly et al 2007 | Intervention | Not include HIIT |
| Wang b | Kim et al 2007 | Intervention | Not include HIIT |
| Wang b | Koh et al 2017 | Intervention | Not include HIIT |
| Wang b | Kolahodouzi et al 2019 | Intervention | Not include HIIT |
| Chang | Ku et al 2010 | Intervention | Not include HIIT |
| Chang | Kwon et al 2010 | Intervention | Not include HIIT |
| Wang b | Lee et al 2010 | Intervention | Not include HIIT |
| Chang | Lesser et al 2016 | Intervention | Not include HIIT |
| Wang b | Lopes et al 2016 | Intervention | Not include HIIT |
| Wang b | Mendham et al 2014 | Intervention | Not include HIIT |
| Wang b | Meyer et al 2006 | Intervention | Not include HIIT |
| Wang b | Mezghanni et al 2014 | Intervention | Not include HIIT |
| Chang | Murphy et al 2012 | Intervention | Not include HIIT |
| Wang b | Nikseresht et al 2014a | Intervention | Not include HIIT |
| Wang b | Nikseresht et al 2014b | Intervention | Not include HIIT |
| Wang b | Nono et al 2020 | Intervention | Not include HIIT |
| Wang b | Olson et al 2007 | Intervention | Not include HIIT |
| Wang b | Paahoo et al 2021 | Intervention | Not include HIIT |
| Chang | Park et al 2003 | Intervention | Not include HIIT |
| Wang b | Park et al 2015 | Intervention | Not include HIIT |
| Wang b | Park et al 2020 | Intervention | Not include HIIT |
| Wang b | Phillips et al 2012 | Intervention | Not include HIIT |
| Chang | Poehlman et al 2000 | Intervention | Not include HIIT |
| Wang b | Racil et al 2016 | Intervention | Not include HIIT |
| Wang b | Shabani et al 2018 | Intervention | Not include HIIT |
| Wang b | Shahram et al 2016 | Intervention | Not include HIIT |
| Chang | Sigal et al 2007 | Intervention | Not include HIIT |
| Wang b | Slamat et al 2016 | Intervention | Not include HIIT |
| Chang | Slentz et al 2005 | Intervention | Not include HIIT |
| Chang | Slentz et al 2011 | Intervention | Not include HIIT |
| Chang | Solis et al 2017 | Intervention | Not include HIIT |
| Alzar-Teruel | Taaffe et al 1999 | Intervention | Not include HIIT |
| Wang b | Tomeleri et al 2016 | Intervention | Not include HIIT |
| Chang | Yan et al 2019 | Intervention | Not include HIIT |
| Wu | Bell et al 2015 | Intervention | Short duration of intervention |
| Wu | Ahmaidi et al 1998 | Outcome | Irrelvant outcome |
| Batacan | Astorino et al 2011 | Outcome | Irrelvant outcome |
| Sultana | Astorino et al 2017 | Outcome | Irrelvant outcome |
| Rugbeer | Baekkerud et al 2018 | Outcome | Irrelvant outcome |
| Sultana | Banitalebi et al 2019 | Outcome | Irrelvant outcome |
| Batacan | Bayati et al 2011 | Outcome | Irrelvant outcome |
| Depiazzi | Bento & Rodacki 2015 | Outcome | Irrelvant outcome |
| Wu | Bouaziz et al 2018b | Outcome | Irrelvant outcome |
| Wu | Bouaziz et al 2019b | Outcome | Irrelvant outcome |
| Sultana | Boutcher et al 2013 | Outcome | Irrelvant outcome |
| Depiazzi | Broman et al 2006 | Outcome | Irrelvant outcome |
| Batacan | Chtara et al 2005 | Outcome | Irrelvant outcome |
| Batacan | Ciolac et al 2010 | Outcome | Irrelvant outcome |
| Batacan | Ciolac et al 2011 | Outcome | Irrelvant outcome |
| Batacan | Croft et al 2009 | Outcome | Irrelvant outcome |
| Sultana | Currie et al 2015 | Outcome | Irrelvant outcome |
| Batacan | Esfarjani et al 2007 | Outcome | Irrelvant outcome |
| Sultana | Foster et al 2015 | Outcome | Irrelvant outcome |
| Wu | Grace et al 2018 | Outcome | Irrelvant outcome |
| Batacan | Guelfi et al 2005 | Outcome | Irrelvant outcome |
| Depiazzi | Hammer and Morton 1990 | Outcome | Irrelvant outcome |
| Batacan | Hood et al 2011 | Outcome | Irrelvant outcome |
| Wu | Hurst et al 2019 | Outcome | Irrelvant outcome |
| Batacan | Keteyian et al 2014 | Outcome | Irrelvant outcome |
| Sultana | Lee et al 2017 | Outcome | Irrelvant outcome |
| Batacan | Leggate et al 20102 | Outcome | Irrelvant outcome |
| Wu | Losa-Reyna et al 2019 | Outcome | Irrelvant outcome |
| Batacan | Macdougall et al 1998 | Outcome | Irrelvant outcome |
| Batacan | Maran et al 2010 | Outcome | Irrelvant outcome |
| Wu | Masuki et al 2017 | Outcome | Irrelvant outcome |
| Sultana | Mazurek et al 2014 | Outcome | Irrelvant outcome |
| Sultana | Mckie et al 2018 | Outcome | Irrelvant outcome |
| Sultana | Mejas-Pena et al 2016 | Outcome | Irrelvant outcome |
| Sultana | Metcalfe et al 2012 | Outcome | Irrelvant outcome |
| Depiazzi | Michaud 1995 | Outcome | Irrelvant outcome |
| Batacan | Molmen-Hansen et al 2012 | Outcome | Irrelvant outcome |
| Depiazzi | Moreira et al 2013 | Outcome | Irrelvant outcome |
| Rugbeer | Rafiei et al 2019 | Outcome | Irrelvant outcome |
| Batacan | Richards et al 2010 | Outcome | Irrelvant outcome |
| Rugbeer | Robinson et al 2015 | Outcome | Irrelvant outcome |
| Batacan | Rustad et al 2014 | Outcome | Irrelvant outcome |
| Wu | Santos et al 2019 | Outcome | Irrelvant outcome |
| Sultana | Schaun et al 2018 | Outcome | Irrelvant outcome |
| Sultana | Scribbans et al 2014 | Outcome | Irrelvant outcome |
| Wu | Sogaard et al 2019 | Outcome | Irrelvant outcome |
| Sultana | Songsorn et al 2016 | Outcome | Irrelvant outcome |
| Sultana | Stavrinou et al 2018 | Outcome | Irrelvant outcome |
| Sultana | Stutnik et al 2016 | Outcome | Irrelvant outcome |
| Batacan | Tabata et al 1996 | Outcome | Irrelvant outcome |
| Sultana | Tanisho & Hirakawa 2009 | Outcome | Irrelvant outcome |
| Sultana | Trilk et al 2011 | Outcome | Irrelvant outcome |
| Sultana | Wilson et al 2019 | Outcome | Irrelvant outcome |
| Wu | Wyckelsma et al 2017a | Outcome | Irrelvant outcome |
| Wu | Wyckelsma et al 2017b | Outcome | Irrelvant outcome |
| Andreato | Bækkerud et al 2016 | Outcome | Missing data |
| Keating | Fisher et al 2015 | Outcome | Missing data |
| Guo | Francois et al 2018 | Outcome | Missing data |
| Steele | Galedari et al 2017 | Outcome | Missing data |
| Guo | Heiston et al 2020 | Outcome | Missing data |
| Wewege | Kemmler et al 2014 | Outcome | Missing data |
| Steele | Martins et al 2016 | Outcome | Missing data |
| Sultana | Matsuo et al 2014b | Outcome | Missing data |
| Steele | Shing et al 2013 | Outcome | Missing data |
| Keating | Thomas et al 1984 | Outcome | Not in full text (abstract only) |
| Wang a | Alberga et al 2015 | Population | Child/ Teenagers |
| Wang a | Barbeau et al 2007 | Population | Child/ Teenagers |
| Steele | Boer et al 2014 | Population | Child/ Teenagers |
| Keating | Buchan et al 2011 | Population | Child/ Teenagers |
| Steele | Camacho-Cardenosa et al 2016 | Population | Child/ Teenagers |
| Wang a | Cao et al 2022 | Population | Child/ Teenagers |
| Keating | Corte de Araujo et al | Population | Child/ Teenagers |
| Wang a | Davis et al 2011 | Population | Child/ Teenagers |
| Wang a | Davis et al 2012 | Population | Child/ Teenagers |
| Steele | De Araujo et al 2012 | Population | Child/ Teenagers |
| Steele | Dias et al 2018 | Population | Child/ Teenagers |
| Steele | Koubaa et al 2013 | Population | Child/ Teenagers |
| Keating | koubba et al | Population | Child/ Teenagers |
| Wang a | Lee et al 2012 | Population | Child/ Teenagers |
| Wang a | Lee et al 2013 | Population | Child/ Teenagers |
| Wang a | Mitchell et al 2002 | Population | Child/ Teenagers |
| Wang a | Monteriro et al 2015 | Population | Child/ Teenagers |
| Steele | Morrissey et al 2018 | Population | Child/ Teenagers |
| Steele | Murphy et al 2015 | Population | Child/ Teenagers |
| Wang a | Saelens et al 2011 | Population | Child/ Teenagers |
| Wang a | Staiano et al 2017 | Population | Child/ Teenagers |
| Steele | Starkoff et al 2014 | Population | Child/ Teenagers |
| Chang | Almenning et al 2015 | Population | Clincial population |
| Andreato | Alvarez et al 2016 | Population | Clincial population |
| Andreato | Alvarez et al 2018 | Population | Clincial population |
| Alzar-Teruel | Ballesta-García et al 2019 | Population | Clincial population |
| Sultana | Boer and Moss 2016 | Population | Clincial population |
| Steele | Boer et al 2016 | Population | Clincial population |
| Wu | Bouaziz et al 2019 a | Population | Clincial population |
| Chang | Cassidy et al 2016 | Population | Clincial population |
| Batacan | Conraads et al 2015 | Population | Clincial population |
| Keating | Devin et al 2016 | Population | Clincial population |
| Steele | Earnest et al 2013 | Population | Clincial population |
| Batacan | Freese et al 2015 | Population | Clincial population |
| Guo | Gilbertson et al 2019 | Population | Clincial population |
| Guo | Gyorkos et al 2019 | Population | Clincial population |
| Chang | Hallsworth et al 2015 | Population | Clincial population |
| Wu | Hwang et al 2019 | Population | Clincial population |
| Sultana | Jung et al 2015 | Population | Clincial population |
| Chang | Karstoft et al 2013 | Population | Clincial population |
| Batacan | Little et al 2011 | Population | Clincial population |
| Batacan | Madssen et al 2014 | Population | Clincial population |
| Steele | Magalhaes et al 2020 | Population | Clincial population |
| Steele | Maillard et al 2016 | Population | Clincial population |
| Steele | Mastuo et al 2015 | Population | Clincial population |
| Sultana | Matsuo et al 2015 | Population | Clincial population |
| Batacan | Mitranun et al 2014 | Population | Clincial population |
| Hwang | Moholdt et al 2009 | Population | Clincial population |
| Batacan | Moholdt et al 2012 | Population | Clincial population |
| Depiazzi | Mohr 2014 | Population | Clincial population |
| Andreato | Morales-Palomo et al 2017 | Population | Clincial population |
| Batacan | Nytroen et al 2013 | Population | Clincial population |
| Steele | Oh et al 2017 | Population | Clincial population |
| Andreato | Ortega et al 2016 | Population | Clincial population |
| Steele | Pasetti et al 2012 | Population | Clincial population |
| Guo | Pedersen et al 2019 | Population | Clincial population |
| Keating | Ramos et al 2016 | Population | Clincial population |
| Sultana | Ramos et al 2017 | Population | Clincial population |
| Hwang | Rodnmo et al 2004 | Population | Clincial population |
| Sultana | Ruffiino et al 2017 | Population | Clincial population |
| Andreato | Stensvold et al 2010 | Population | Clincial population |
| Andreato | Stensvold et al 2012 | Population | Clincial population |
| Batacan | Terada et al 2013 | Population | Clincial population |
| Hwang | Tjonna et al 2008 | Population | Clincial population |
| Sultana | Toohey et al 2018 | Population | Clincial population |
| Depiazzi | Waller 2017 | Population | Clincial population |
| Steele | Winding et al 2018 | Population | Clincial population |
| Chang | Winn et al 2018 | Population | Clincial population |
| Hwang | Wisloff et al 2007 | Population | Clincial population |
| Rugbeer | Barry et al 2018 | Population | mixing clincial and non-clinical |
| Guo | Sartor et al 2010 | Population | mixing clincial and non-clinical |
| Sultana | Alvarez et al 2012 | Study | Language (not in English) |
| Andreato | Escarate et al 2016 | Study | Language (not in English) |
| Andreato | Hormazábal et al 2016 | Study | Language (not in English) |
| Batacan | Astorino et al 2012 | Study | Non randomized design |
| Batacan 2017 | Dupont et al 2004 | Study | Non randomized design |
| Batacan | Tremblay et al 1994 | Study | Non randomized design |
| Sultana 2019 | Yamagishi and Barbaj 2017 | Study | Non randomized design |
